# Supplementary material for: ‘Well London’ and the benefits of participation: results of a qualitative study nested in a cluster randomised trial
Source: BMJ Open. 2014 Apr 1;4(4):e003596. doi: 10.1136/bmjopen-2013-003596 (PMC3987724; doi:10.1136/bmjopen-2013-003596)
Supplement: Author's manuscript [file bmjopen-2013-003596.draft_revisions.pdf]

'Well London' and the benefits of participation: results of a qualitative study nested in a cluster randomised trial

Journal:	*BMJ Open*
Manuscript ID:	bmjopen-2013-003596
Article Type:	Research
Date Submitted by the Author:	16-Jul-2013
Complete List of Authors:	Derges, Jane; University of Westminster, Life Sciences<br>Clow, Angela; University of Westminster, Psychology<br>Lynch, Rebecca; University of Westminster, Life Sciences<br>Jain, Sumeet; University of Westminster, Life Sciences<br>Phillips, Gemma; University of East London, Institute for Health and Human Development<br>Petticrew, Mark; London School of Hygiene and Tropical Medicine, Public Health Evaluation<br>Renton, Adrian; University of East London, Institute for Health and Human Development<br>Draper, Alizon; University of Westminster, Life Sciences
**Primary Subject Heading**:	Public health
Secondary Subject Heading:	Qualitative research
Keywords:	PUBLIC HEALTH, QUALITATIVE RESEARCH, SOCIAL MEDICINE

SCHOLARONE™ Manuscripts

'*Well London*' and the benefits of participation: results of a qualitative study nested in a cluster randomised trial

Corresponding author:

Dr Jane Derges, School of Life Sciences, University of Westminster, 115 New Cavendish Street, London W1W 6UW

dergesj@westminster.ac.uk

Phone: 020 7911 5000 ext. 3884

Fax: 020 7911 5087

Co-authors:

Prof. Angela Clow, Psychology Department, University of Westminster, London, UK

Ms Rebecca Lynch, School of Life Sciences, University of Westminster, London, UK

Dr Sumeet Jain, School of Life Sciences, University of Westminster, London, UK

Dr Gemma Phillips, Institute for Health and Human Development, University of East London, UK

Prof. Mark Petticrew, Department of Public Health, London School of Hygiene and Tropical Medicine, London, UK

Prof. Adrian Renton, Institute for Health and Human Development, University of East London, UK

Dr Alizon Draper, School of Life Sciences, University of Westminster, London, UK

Public health, programme evaluation, qualitative study
Word count: 3129

For peer review only

ABSTRACT

Background

Well London is a multi-component community engagement programme designed to improve the health of Londoners living in socio-economically deprived neighbourhoods. To evaluate outcomes of the *Well London* interventions, a cluster randomized trial (CRT) was conducted that included a longitudinal qualitative component. The aim was to explore in depth the nature of the benefits to residents and the processes by which these were achieved.

Methods

The one-year longitudinal qualitative study was nested within the CRT. Purposive sampling was used to select three intervention neighbourhoods in London and 61 individuals within these neighbourhoods. Interviews were conducted at inception and following completion of the *Well London* interventions. Transcripts of the interviews were coded and analysed using Nvivo.

Results

Positive benefits relating to the formal outcomes of the CRT were reported, but only among those who participated in project activities. The extent of benefits experienced was influenced by factors relating to the physical and social characteristics of each neighbourhood. The highest levels of change occurred in the presence of: a) social cohesion, pre-existing but also as facilitated by *Well London* activities; b) personal and collective agency; c) involvement and support of external organisations. Where the physical and social environment remained unchanged, there was less participation and fewer benefits.

Conclusion

These findings show interaction between participation, well-being and agency, social interactions and cohesion, and that this modulated any benefits described. Pathways to change were thus complex and variable, but both personal well-being and local social cohesion emerged as important mediators of change.

Article summary

Article focus

- To show if a neighbourhood-based health intervention programme changed individual health and well-being.
- To show whether the use of a community engagement approach enhances the uptake and impact of project activities.

Key messages

- Individual well-being and agency are critical to encouraging participation
- Well-being is a key mediator of change in health practice
- Improving social capital/cohesion through exercising personal agency leads to 'well-being', which results in participation

Strengths and limitations of this study

- Social capital/cohesion is efficacious for community-based health programmes Community engagement is vital to reducing health inequalities, but questions remain about how it influences health outcomes and how to achieve inclusive participation
- In depth analysis of non-participation is a future and potentially important research topic

For peer review only - http://bmjopen.bmj.com/site/about/guidelines.xhtml

INTRODUCTION

Processes of change in community-based interventions

Improving the health and well-being of populations living in disadvantaged areas of the UK remains a key public health challenge[1,2]. But while there is extensive evidence documenting the consequences and causes of health inequalities, less is known about the interplay between specific causal factors or what interventions are effective in reducing them[3]. There is growing recognition of the need to understand which interventions are effective and the processes or pathways by which effects are achieved[4-7]. This is particularly important for interventions that are "complex"[8,9] and in which local contextual factors modulate both the process of implementation and generation of outcomes[10]. The *Well London* programme is a complex intervention comprising multiple components and using a community engagement model. The interventions comprised a series of activities based around healthy eating, physical activity and mental well-being. 'Well-being' is here defined as a eudemonic state in which the individual experiences positive engagement, a sense of meaningfulness and usefulness in life. This framework was used in each area but the delivery method varied according to local needs and priorities, as outlined in current theories concerning the design and evaluation of complex interventions[11,12]. Further details are obtainable from the *Well London* Website[13].
As Draper at al. and others note there is a need for rigorous evaluations that explore the causal pathways by which community engagement influences health outcomes[14-16]. Popay[17] and Wallerstein[18] have hypothesized a number of possible pathways, but these remain largely unexamined. The relationship between social context, individual agency and participation has also been neglected[19], as well as exploration of the psychosocial mechanisms and effects of interventions to address health inequalities[20] and the nature of personal agency in a social determinants of health framework[21]. Whilst autonomy in decision-making is recognized as having a positive role, there is little in the way of qualitative studies that examine agency in relation to community engagement programmes.

These issues are explored here using data from a qualitative study embedded within the cluster randomized trial (CRT) of the *Well London* programme (see[22] for a full description of the trial protocol). The primary aim of the qualitative study was to examine the causal pathways that generated any intervention effects from the perspectives of local residents, who were involved as strategic partners in *Well London*'s design and delivery.

METHODS

Study Objectives

The objectives of the qualitative study were:

1. To identify the different ways in which individuals became engaged and continued to participate in *Well London* activities;
2. To explore how the different project components helped enable people to improve their health and health practices;
3. To identify factors in the social and physical environment that shape attitudes to health.

Study design

An in-depth qualitative research element was included in the CRCT in order 1) to address the complexity[23] of the intervention; 2) to try to identify specific factors which enable or obstruct individuals in leading healthy lives; 3) to understand subjective experience and the role of 'agency' in relation to participation and engagement.

For peer review only

The longitudinal qualitative study component was nested within the larger *Well London* CRT (for details of overall trial design, see[24]). It comprised a series of in depth interviews conducted over a period of one year, with interviews undertaken in two stages: firstly, at the implementation stage of the interventions and secondly, post-intervention. Both participants and non-participants of *Well London* were interviewed to capture whether exposure to the interventions would lead to neighbourhood level improvements in health and health practices, or whether direct participation was required. Limited observation of three selected *Well London* intervention neighbourhoods was also undertaken, to contextualise the interview data.

Selection of study neighbourhoods

Initial observation showed contextual variation between the twenty *Well London* intervention neighbourhoods in environment, demography and history as well as in intensity and range of community activities running parallel to (i.e not commissioned by) *Well London*. A critical case sampling approach was therefore used to select 3 neighbourhoods to be included in the qualitative study (chosen in consideration of the in depth nature of the study). This approach selects cases based on criteria that are seen to be particularly important for the research project: “if it happens there, it will happen everywhere” or “if it doesn’t happen there it won’t happen anywhere”, “if that group is having problems we can be sure that every group is having problems”[25].

In selecting this approach it was necessary to identify what would make a critical case in relation to the objectives of the qualitative study. We therefore included neighbourhoods with both low and high levels of community projects. The method of programme delivery within *Well London* also differed and the three neighbourhoods chosen reflect this by including neighbourhoods with high and low levels of pre-existing community activities beyond those provided by the *Well London* programme, and differences in the manner of their delivery.

The first of these neighbourhoods (Eastford[1]) had a wide range of community activities offered prior to *Well London*, and continues to offer many activities unconnected to *Well London*. The second neighbourhood (Hartfield) has a core group of volunteers instrumental in generating engagement around *Well London* activities and they live in an enclosed geographical space (a housing estate). The third neighbourhood (Mountside) had limited community activities prior to *Well London* and a population dispersed among a number of differing housing sites. The manner of *Well London* delivery in these neighbourhoods varied from highly pro-active and involved members to a less cohesive and active method of delivery.

Study population

61 individuals were recruited at the start of *Well London* delivery and comprised matched participants of the interventions, and non-participants (see Table 1). Participants were purposively selected from within the interventions across the 3 study neighbourhoods and non-participants were selected through snowball sampling; these contacts were made by the researcher during visits to the neighbourhood. ‘Participant’ is here defined as residents who both received the *Well London* activities, and volunteered in their delivery.

For the post-intervention interview, a total of 45 agreed to a second interview. Reasons given by the 16 who did not attend this second interview were as follows: moved out of the neighbourhood (2), refusal of a follow-up interview (3), no response elicited (9), and illness (2). New recruits were not sought as changes over the intervening period would not have been captured.

Ethnicity, age and length of time in the neighbourhood among the study population were mixed across all three neighbourhoods; each neighbourhood showed variation according to all

[1] Pseudonyms are used throughout for places and people

For peer review only

these categories, most noticeably, ethnicity. It is beyond the scope of this paper to examine the effects of this in detail, other than to recognize this as a difference requiring further investigation.

Table 1. Participant and non-participant profiles

1st Round interviews	Age range	Ethnicity	Gender	*Well London* participation	2nd Round interviews
Hartfield	16-25: 3<br>26-35: 8<br>36-45: 3<br>46-55: 5<br>56-65: 2<br>66-89: 1	African 13, Indian 3, Bangladeshi 1, White British 3, European (Lithuania) 1	Female: 16<br>Male: 5	Participants: 13<br>Non-participant: 8<br><br>Total: 21	Stage 2.<br>P=11<br>NP=8<br><br>Total: 20
Eastford	16-25: 4<br>26-35: 4<br>36-45: 2<br>46-55: 4<br>56-65: 3<br>66-75: 3	Bangladeshi 5, Indian 1, Pakistani 1, Caribbean 2, Black British 1, African 3, Chinese 2, White British 3, Irish 2	Female: 18<br>Male: 2	Participants: 11<br>Non-participants: 9<br><br>Total: 20	Stage 2.<br>P=7<br>NP=8<br><br>Total: 15
Mountside	16-25: 5<br>26-35: 3<br>36-45: 4<br>46-55: 3<br>56-65: 3<br>66-75: 2	White British 5, British Asian 5, Caribbean 4, European (Turkish) 3, African 2, Chinese 1	Female: 11<br>Male: 9	Participants: 10<br>Non-participant: 10<br><br>Total: 20	Stage 2.<br>P=6<br>NP=4<br><br>Total: 10

Data collection and analysis

Pre- and post intervention interviews used the same topic guide and focused on participants experiences of the *Well London* interventions and any reported changes to eating, exercise and mental health practices (see supplementary file). Also both participants and non-participants were asked for their views about the neighbourhood environment. Interviews were recorded and transcribed before being entered into Nvivo. Each transcript was checked for quality, coded and analysed using a framework based on Spencer, Ritchie and O'Conner's 'analytic hierarchy'[26]). This allowed systematic analysis of the large dataset but was flexible enough to allow refinements to the coding. Codes from interviews were identified and grouped under categories generated from the interview topics. Data were analysed not only deductively from the primary outcome measures (changes to healthy eating, physical activity and mental well-being), but also inductively from emerging themes identified from the interviews. Observational data of the neighbourhoods was recorded both in photographs and written notes and included the local geography, amenities and range of community facilities and activities that were available. A separate researcher was employed at each stage of the study; one researcher for stage 1 interviews, and the second researcher for stage 2. They each also conducted the observations simultaneous to the interviews.

Interview quality assurance and ethics

Quality assurance procedures were undertaken to minimise researcher bias when coding the interviews by randomly selecting three interviews, which were then recoded by two independent researchers blind to the initial coding. The three interviews were compared to identify new codes and establish a degree of consensus in applying a particular code to similar

For peer review only - http://bmjopen.bmj.com/site/about/guidelines.xhtml

text. The University of Westminster Research Ethics Committee approved the study. Following initial telephone/email contact, written consent was obtained from every participant/non-participant. Each individual also received a short pamphlet describing the *Well London* project and an explanation of the qualitative component. Verbal explanation was also provided at the start of the interview process.

The results of the qualitative study are presented here by area. The reason for this is that the context and environment in which participants and non-participants were living and into which the *Well London* interventions and activities were introduced, has been shown to be a key factor in showing why individuals participated. Subsequently, data are presented by area, not theme.

RESULTS

Findings from the qualitative data show that participants described positive changes to both individual health and social cohesiveness within the neighbourhood as a result of participation in the *Well London* activities. As participation was key in engaging individuals, equally significant was the impact of neighbourhood variations, which were modulated by: mode of delivery, characteristics of individuals, neighbourhood history and attitudes to social interaction.

Participants identified the combination of activity alongside social interaction as crucial to engagement. For example: healthy food introduced as part of a social gathering; gardening followed by chatting over a cup of tea; women enjoying an evening walk together. These were experienced by participants as having a key beneficial effect within the more practical elements of an activity. By comparison, non-participants, despite their individual attempts at improving their own health, experienced no benefits either from efforts to change eating or physical activity levels, or from being around others in the community who were participating in *Well London* activities:

> ‘*So I was left a very lonely bunny for quite a while. I don’t like going out walking all the time on my own, I don’t like going swimming on my own. I love to do it, but I don’t like doing it on my own. If there was a group going, I would go’* Mary, age 48, Irish, non-participant

Furthermore, a small number of non-participants felt excluded from the *Well London* interventions, suggesting there may be some non-beneficial effects. For example, in response to a question about positive changes on the estate as a result of *Well London*, one resident commented:

> ‘*Well, that “getting better” is a matter of opinion, because I look on it now as a ghetto. It was an unruly estate before. It has quietened down, but now it’s a ghetto. I don’t go out anymore, I don’t do anything anymore. No. No. And I hardly even talk to people now. I mean, I’ll sit out at my doorstep and, you know, a lot of people’ll stop and chat to me, but I don’t really like it’.* Karen, age 54, White British, non-participant

Despite scoring high on the ‘Indices of Deprivation’[27], the three *Well London* intervention neighbourhoods will be described separately in acknowledgement of their diversity and to bring out the nuances of how place impacts participation and any consequent outcomes.

HARTFIELD is a large housing estate built in the 1950s, comprising low-rise blocks constructed around a series of rectangular grassed areas. Although the most homogenous of the three

neighbourhoods in terms of population and environment, prior to *Well London* it was socially fragmented with a dearth of community activities.

Pre-intervention descriptions of Hartfield included:

> *'And the word 'Hartfield' put horror – it was notorious. Everybody who was difficult was dumped here'.* Margaret, 59, White British

> *It's a dumping ground. It always has been a dumping ground. You know? I begged not to be put on there. I've been there 21 years.* Liz, 48, Irish

> *I will tell you straightaway there was no life in the community before the arrival of* Well London*. No, that is the summary of the whole thing; where you are living in an area where there was no life.* Clifford, 46, African (Uganda)

Post-intervention, Hartfield respondents reported the most substantive change in experienced health benefits of all 3 neighbourhoods. Factors that facilitated this included: a) a pro-active, charismatic Well-London coordinator; b) increased safety following changes in policing methods on the estate, instigated by the coordinator; c) a high number of proactive volunteers; and d) residents as stakeholders through the estate's Residents Committee, set up by *Well London*. Benefits described included: enhanced feeling of social cohesion, new knowledge about health, involvement in estate-wide activities, improved relations with neighbours, less complaints about the neighbourhood's lack of cleanliness, safety and violence.

Box 1 Hartfield	Reported benefits in HE, PA, MHWB, and Social Interaction
**Healthy Eating**	➢ *So you know, after walk we have this exercise to stretch ourselves, and then after that we used to have fruit. Yeah, so we used to sit in the park and we used to eat fruit and that's how I learn to eat fruit basically.* Priya, 34, Indian<br>➢ *Oh my God, people are healthier now. It's changed, it's completely changed. I say that it's changed because I am involved - I know how much to my own particular health* (and*) the health of my family and how much has changed. I'm able to know more now, I know what to eat, what not to eat.* Thomas, 45, African (Ghana)
**Physical Activity**	➢ *Like before, you know, I used to find walking was kind of one of the painful things, yeah, I wouldn't bother to walk, I would rather take bus rather than walking, but now I feel like, now - rather than taking bus or anything, let's just walk, it's not going to take me that long.* Sandra, 43, African (Uganda)<br>➢ *And we have some people who want to go night walk - like the Somalians. If the place is dark, they would like to walk. Because of, you know, night-time you can also wear your trousers - so that they can walk faster.* Joyce, 38, African (Nigeria)

**Mental Health, Wellbeing**	➢ *The fact that it's made me proud of myself and the whole project and the whole community, because it's made people come in to do the activities.* Bernard 42, African (Ivory Coast)<br>➢ *It has made the community come together, that's what I've seen anyway, people have come together, which is very good.* Claudette, 37, African (Sierra Leone)
**Social interaction**	➢ *Yes we used to be on our own, nobody say hello to each other, but because of Cheryl,* Well London *came to this place* (and*) it start connecting us.* Lorraine, 39, African (Uganda)<br>➢ *Yeah the police, which are responsible to this area, yes it's changed a lot because now they can say 'hello' to you. Sometimes even the kids, if they see them playing outside they will stand and speak with them, and ask them 'are you with elderly adults or are you alone?' And so forth and we are happy for that.* Margaret, 41, African (Ghana)<br>➢ *I think that it does a good thing -* Well London *came to help out. We did a food basket with five foods, I did that as well so. I know the women come to do, they have sewing classes, and it's just - but it's for the coming together, the community together, that's what I think.* Clara, 42, Bangladeshi<br>➢ *I've been proud to say that this is one of my proudest periods in regards to this community. Yeah, this is because our efforts that has been put in place by* Well London *and followed by* Well London *volunteers. The Hartfield estate now compared to what it was in the past, it's a name at least to be proud of.* Frank, 39, African (Nigerian)

EASTFORD has undergone extensive regeneration over the last decade, including funding to develop community projects that promote health. This had generated an ethos of community engagement and differentiating the *Well London* interventions from these other activities was subsequently more difficult, especially its effects on Mental Health and Well-being. Despite this, the positive changes experienced here by participants refer specifically to the *Well London* activities.

Pre-intervention, Eastford was already defined as a place where things happened: 'Eastford *is great – there's so much to do here!'* Jermina, 26, Bangladeshi.
Post-intervention, respondents experienced some change. Benefits included a) a sense of autonomy from volunteering and involvement in managing and running activities; b) feeling productive and useful; c) increased knowledge of food/cooking & improved health; and c) enhanced feeling of social cohesion.

**Box 2 Eastford**	**Reported benefits in HE, PA, and MHWB and Social Interaction**
**Healthy Eating**	➢ *Yeah, whereas say somebody comes in on Tuesday and does a little bit of cooking - it's quite quick, but with the 'Cook & Eat' it was more in depth and they explained things better and you could ask questions and all things like that, yeah, it was much better.* Clare, 38, White British<br>➢ *Earlier I used to be like, junkie foods eating; crisps and all*

	those things. Now it's like more fruit and vegetables and salad in my diet. Shubha, 28, Indian
**Physical Activity**	➢ *It was great, it's fantastic - I cannot express how good it is to get in there and get your hands dirty, and to see everybody else doing the same thing.* Sarah, 34, White British<br>➢ *Yes, I do a lot, because I'm doing them exercises it's helped me, it's good for my health, I feel much better, I can breathe properly. And you make friend. Yeah, it's good for me - I go out, and you meet friends.* Tricia, 72, Caribbean
**Mental Health and Well-being**	➢ *I feel I can keep my mind going and I feel like my mind has to be active because I don't want to sit down and get depressed or something. If I think bad things then I won't be doing nothing and I don't want to go like that yeah.* Maureen, 48, Irish
**Social interaction**	➢ *I feel so much more confident that we can make this move on; the thing we were given was confidence building. I think that sort of confidence building was something I didn't see – yeah, running an organisation, running that level of budgeting and planning.* Michael, 50, White British<br>➢ *You can see it, just a healthier lifestyle: people busy all the time, people - not so much arguments and you see that less and people are a lot more sociable as well.* Pat, 36, Black British

MOUNTSIDE

Mountside is a neighbourhood of contradictions, characterized by a geographically dispersed, ethnically and socio-economically diverse but transitory population and a reverse trend in terms of regeneration:

> *'It was a transient population so you'd get people move in for three months, as I say, trash the place or do whatever'*. Paula, 45, White British

> *'It became what I can only describe as a dumping ground for literally anybody. There was no perspective on who was living where and next to whom; people were just thrown into the flats regardless of background, criminal intention or anything'*. Mohan, 52, Indian

> *'When we first moved here it was gorgeous. Oh, you couldn't have wished for a more idyllic place to live. It was quiet, it was flowers, it was lovely neighbours. But of course, a lot of our neighbours then had been here since the block went first up in the '60s, so they were all getting old and consequently all started to die and then their families sold the flats to housing associations. And you go from there'*. Monica, 62, White British

The loss of facilities such as a local cinema, shops and other community activities resulted in the main street consisting of fast food outlets and budget shops – high street brands that used to exist had moved away, apart from a large supermarket. There were pockets of privately owned

For peer review only - http://bmjopen.bmj.com/site/about/guidelines.xhtml

terraced housing divided from local authority tower blocks, marking a clear socio-economic boundary. Attitudes to *Well London* were similarly divided; some viewed the interventions positively, and some not.

Post-intervention, Mountside respondents recounted little change; positive change was commented on only in relation to the Mental Health and Well-being activities. Factors that prevented change were: a) lack of effective, coordinated local leadership; b) dispersal and transience of the local population; c) lack of cohesive environmental planning; and d) strong sense of neglect and 'being forgotten' by residents.

**Box 3 Mountside**	**Reported benefits in HE, PA, and MHWB**
**Healthy Eating**	➢ *Yeah, you know children like chips, sausages, yeah. Just sometimes I'm cooking chips - every time Turkish foods; rice yeah. You know, my older one all the time she wants outside, McDonalds, chicken, chips, she's eating too much. And everywhere this food. I'm telling her 'you know, too much oily inside, you no eat' and she's not listening to me.* Hanife, 36, Turkish<br>➢ *You can see the higher fast food intake, zero exercise, high alcohol and stressful kind of lifestyles that people lead. And this is also supported by the number of fast food outlets that thrive in these areas.* Mohan, 31, Asian British
**Physical Activity**	➢ *We've got one park over the road but, again that's a dangerous place. We've had murders over there, we've had people killing the swans to eat and people sleeping rough over there. So of course, parents weren't taking their kids over to the park, and you can't blame them, I wouldn't go over there.* Marie, 46, White British
**Mental Health and Well-being**	➢ *It was fantastic, it brought up a lot of issues and a lot of practices and things that I'm already aware of, and I really, really enjoyed it'.* Saroja, 27, Asian British<br>➢ *Oh yeah, and I wish it could continue, I really, really do, because I think it's started to actually break down a few barriers. We were all really sad when it ended and I thought; this is something that could really build up. And I just wish we could have* Well London *permanently. It was a really nice thing, and because it came to this area it made us think, well we are important, it's come here. I know it came here because we were a deprived area but people are listening to us. They're trying to do something to help us. And like I say, the worst thing is that we haven't got it* (now). *If you can bring it back I'd be ever so grateful and so would a lot of other people.* Karen, 41, White British<br>➢ *I came away having learnt a lot more about the other women – appreciating them more, yeah, I think that's word should be put in there; appreciating other people, not just cultures but people themselves*. Molly, 45, Caribbean
**Social interaction**	➢ *It takes the form of exercise when I can be bothered. I will say I'm a bit lazy sometimes, so you'll do it and then it's like you*

don't want to take it on, on your own, so you do need motivation Jan, age 36, White British

CONCLUSION

Participants described an overall positive impact from the *Well London* project activities, but the data also reveal a complex and nuanced picture of if and how outcomes were achieved with two key findings. Firstly participation was crucial and shows how neighbourhood-level changes did not lead to benefits amongst those who did not participate in project activities. Secondly, the characteristics of neighbourhoods, both social and physical, were fundamental in moderating whether people participated, the nature and extent of the consequent benefits, and any reported changes in health practices.

Well-being and a related sense of personal agency were required to enable participation in the *Well London* activities. Once engaged there was an apparent feedback loop whereby further enhancement of well-being and increased personal agency lead to increased involvement in the activities and changes in attitudes and practices in eating, exercise and mental health. Participants' well-being, agency and participation also interacted with their sense of place, again in an iterative fashion. When people took part in activities the social interaction involved, served to enhance social cohesion and collective agency. Following improvements to the physical environment, further enhancement of well-being and agency were described. Well-being was in this instance a crucial mediator between participation, agency and improved health practices. A recent review of individual experiences of community engagement also found that active engagement in community initiatives has important psychosocial benefits for participants that include enhanced feelings of personal confidence and self-esteem, as well as enhanced social relationships and social cohesion within a community[28, 29].

Participation was not a simple binary variable and quantitative measures alone did not pick up the subtleties and complex variations. Our findings show that participation is a complex and dynamic process with well-being at its core, acting as a catalyst that enables participation through a related sense of personal agency. Through further enhancement of well-being and associated social cohesion, improvements in health practices were experienced, just as in its absence no benefits were recounted.

Neither are health practices a separate 'capsule' of behaviour[30, 31], but are embedded within particular social, cultural and physical milieus. This is illustrated by the number of *Well London* participants who spoke positively about the impact of the interventions and activities in creating a socially more cohesive atmosphere. Across the three neighbourhoods however, there was a clear gradient of change with the greatest change seen in the presence of; a) social cohesion fostered by *Well London* activities; b) personal and collective agency enhanced by involvement in project activities; c) involvement and support of external organisations. Where the physical and social environment remained unchanged, there was less participation and therefore fewer benefits.

As others have identified, the dynamics of participation from the perspective of individual agency have been neglected[17, 18, 29]. In addressing this, our findings show participation as a dynamic and flexible process with agency at its core. Also, the community engagement approach fed back into and reinforced feelings of well-being and agency and thus encouraged and supported changes in health practices. The findings are consistent with elements of Popay's[17] proposed pathways by which community engagement leads to health outcomes, and specifically that social capital/cohesion and community empowerment are important

For peer review only - http://bmjopen.bmj.com/site/about/guidelines.xhtml

intermediaries[17]. Additionally they point to the need for further research to understand how these interact with agency, well-being and empowerment at the individual level and in different social contexts, in order to achieve a more inclusive engagement with such programmes.

Acknowledgements We thank all of the study participants for giving generously of their time; the project delivery partners for their assistance; our colleagues at the University of East London, University of Westminster and the London School of Hygiene and Tropical Medicine for their support and cooperation.
Author contributions: J Derges data collection, interpretation, analysis and writing; S Jain, data interpretation and analysis; A Clow, data analysis and interpretation; R Lynch, data collection, data analysis; A Renton, project lead; A Draper, data interpretation, analysis and writing.
Funding: This work was supported by the Wellcome Trust, Grant code: 083679/Z/07/Z
Competing interests: None
Ethics approval: granted by University of Westminster Ethics Committee
Data sharing statement: Anonymised interview transcripts may be obtained on request from Dr A. Draper, University of Westminster

REFERENCES

1. WHO. The Jakarta Declaration on Leading Health Promotion into the 21st Century. Fourth International Conference on Health Promotion, Jakarta, 1997.
2. Marmot M. The Marmot Review: Fair Society, Healthy lives – Strategic Review of Health Inequalities in England post-2010. London, 2010.
3. Whitehead M. A typology of actions to tackle social inequalities in health. Journal of Epidemiology and Community Health 2007;**61:**473-478.
4. Anderson A. The Community Builder's Approach to Theory of Change: A Practical Guide to Theory Development. New York: The Aspen Institute Roundtable on Community Change 2005.
5. Bryce J, Victora CG, Habicht JP, Black RE, et al. (MCE-IMCI Technical Advisors) Programmatic pathways to child survival: results of a multi-country evaluation of Integrated Management of Childhood Illness. Health Policy and Planning Suppl 2005;**1:**15-117.

For peer review only - http://bmjopen.bmj.com/site/about/guidelines.xhtml

6. O'Dwyer LA, Baum F, Kavanagh A, MacDougall C. Do area-based interventions to reduce health inequalities work? A systematic review of evidence. Critical Public Health, 2007;**17**:317-335.

7. Victora CG, Habicht J-P, Bryce J. Evidence-based public health: moving beyond randomized trials. American Journal of Public Health 2004;**94**:400-405.

8. Medical Research Council A framework for development and evaluation of RCTs for complex interventions to improve health London: MRC; 2000.

9. Medical Research Council Developing and evaluating complex interventions: new guidance. London: MRC; 2008.

10. Hawe P , Sheill A, Riley T, Gold, L. Methods for exploring implementation variation and local context within a cluster randomized community intervention trial. Journal of Epidemology and Community Health 2004;**58**:788-793.

11. Ogilvie D, Cummins S Petticrew M, White M, Jones A, Wheeler K. Assessing the evaluability of complex public health interventions: five questions for researchers, funders, and policymakers. Milbank Q 2011,**89**:206-225.

12. Hawe P, Shiell A, Riley T. Complex interventions: how "out of control" can a randomized controlled trial be? BMJ 2004, **328**: 1561-1563

13. Well London: communities working together for a healthier city. [http://www.welllondon.org.uk]

14. Draper AK, Hewitt G, & Rifkin SB. Chasing the Dragon: developing indicators for the assessment of community participation in health programmes. Soc Sci Med 2010,71:1102-1109. DOI 10.1016/j.socscimed.2010.05.016

15. Milton B, Attree P, French B et al. The impact of community engagement on health and social outcomes: a systematic review. Community Development Journal 2011;**47**(3): 316-334.

For peer review only

16. Preston R, Waugh H, Larkins, S, Taylor, J. Community participation in rural primary health care: intervention or approach? Australian Journal of Primary HealthCare 2010;**16**:4-16.

17. Popay J. Community engagement and community development and health improvement: a background paper for NICE. London: NICE 2006.

18. Wallerstein N. What is the evidence on effectiveness of empowerment to improve health? Copenhagen, Denmark, WHO Regional Office for Europe, 2006. Health Evidence Network report; http://www.euro.who.int/Document/E88086.pdf. (accessed January 2013)

19. Cleaver F. Institutions, agency and the limitations of participatory approaches to development. In: Hickey S, Mohan G, eds. Participation: From Tyranny to Transformation? London Zed Books, 2004:271-283.

20. Egan M, Tannahill C, Petticrew M, et al. Psychosocial risk factors in home and community settings and their associations with population health and health inequalities: a systematic meta-review. BMC Public Health 2008;**8**:239

21. Forde I, Raine R. Placing the individual within a social determinants approach to health inequity The Lancet 2008;**122**(11):1177-1187.

22. Wall M, Hayes R, Moore D, Petticrew M, et al. Evaluation of community level interventions to address social and structural determinants of health: a cluster randomised controlled trial. BMC Public Health 2009;9:207 DOI:10.1186/1471-2458-9-207.

23. Petticrew M. When are complex interventions 'complex'? When are simple interventions 'simple'? Euro J Public health 2011;**21**:397-8.

24. Phillips G, Renton A, Moore DG et al. The Well London program – a cluster randomized trial of community engagement for improving health behaviors and mental well-being: baseline survey results. Trials 2012;**13**:105.

For peer review only

25. Patton MQ. Qualitative Research and Evaluation Methods. 3rd Edition. Sage Publications, Thousand Oaks, California 2002:236.

26. Spencer L, Ritchie J, O'Connor W. Analysis: Practices, principles and processes. In J. Ritchie & J Lewis, eds. Qualitative research practice: a guide for social science students and researchers. Thousand Oaks, CA: Sage 2003:200-218.

27. English indices of deprivation 2010 Government Report http://www.communities.gov.uk/publications/corporate/statistics/indices2010

28. Clow, A Hamer, M. The iceberg of social disadvantage and chronic stress: implications for public health. Neurosci Biobehav Rev 2010;**35**:1.

29. Attree p, French B, et al. The experience of community engagement for individuals: a rapid review of the evidence. Health and Social Care in the Community 2011;**19**:250-60.

30. Polgar S. Health and human behaviour: areas of interest common to the social and medical social sciences Current Anthropology 1962;**3**:159-205

31. Fienieg B, Nierkins V, Tonkens E, Plochg T, Stronks K. Why play an active role? A qualitative examination of lay citizens' main motives for participation in health promotion. Health Promotion International 2012;**27**:416-26.

For peer review only

UoW WL Evaluation
Revised Interview Topic Guide
18/02/10

UoW Well London Evaluation
Topic Guide for Phase 2 Interviews

Neighbourhood

- **Can you tell me about this area?**
  - What is it like living here?
  - Do you like living here?
  - Who lives here?
  - What are your neighbours like?
  - How did you come to be living in this area?

- **How long have you been here?**
  - Where were you before?
  - How has this area changed since you've lived here?
  - What sense of belonging or attachment, if any, do you feel to the area?
  - Do you see this area as "home"?

- **Is there a lot going on in this area?**
  - What goes on?
  - Who goes?
  - Why do people go/not go?
  - What, if anything, do you participate in in the local area or community?
  - Why do, or why don't you participate in activities in the local area?
  - Do you socialise/have friends locally?

- **How safe and comfortable do you feel in this area?**
  - What are the good things about living here?
  - Do you worry about the area?
  - Have you experienced any problems with the area or the local community?

- **How healthy are people around here?**
  - Are people locally concerned about their health?
  - Is it easy to be healthy here? Do you have access to health activities?
  - Are there things about living in this area that you think are unhealthy?
  - How do you think living in this area affects your health and well being?

Health and Wellbeing

- **What are the features to being healthy?**
  - What is a healthy lifestyle?
  - Would you say you are in good health?
  - Would you say you have a healthy lifestyle?

For peer review only

- **Do you think about your health very much?**
    - What, if anything do you do to keep yourself healthy or improve your health?
    - Do you worry about your health?
    - What do you do about this worry about your health?

- **How much do you worry about things generally?**
    - Do you have a lot to worry about?
    - What do you do when you worry?
    - How optimistic or negative do you think you are as a person?
    - How does this outlook affect the way you live your life do you think?
    - What are the positive and negative things in your life?

- **Are there people you can trust and talk to if you have problems or worries?**
    - Do you have much contact with these people?
    - Do they live nearby?

<u>Well London</u>

- **Have you heard of the Well London Project or know of any Well London activities in the area?**

- **Have you participated in any Well London activities (including the community cafes)?**
    - If so, what was your experience of these?

- **Have you felt there has been any benefit to yourself or your area from the Well London Project?**

For peer review only

'Well London' and the benefits of participation: results of a qualitative study nested in a cluster randomised trial

Journal:	*BMJ Open*
Manuscript ID:	bmjopen-2013-003596.R1
Article Type:	Research
Date Submitted by the Author:	04-Dec-2013
Complete List of Authors:	Derges, Jane; University of Westminster, Life Sciences<br>Clow, Angela; University of Westminster, Psychology<br>Lynch, Rebecca; University of Westminster, Life Sciences<br>Jain, Sumeet; University of Westminster, Life Sciences<br>Phillips, Gemma; University of East London, Institute for Health and Human Development<br>Petticrew, Mark; London School of Hygiene and Tropical Medicine, Public Health Evaluation<br>Renton, Adrian; University of East London, Institute for Health and Human Development<br>Draper, Alizon; University of Westminster, Life Sciences
**Primary Subject Heading**:	Public health
Secondary Subject Heading:	Qualitative research
Keywords:	PUBLIC HEALTH, QUALITATIVE RESEARCH, SOCIAL MEDICINE

SCHOLARONE™ Manuscripts

'*Well London*' and the benefits of participation: results of a qualitative study nested in a cluster randomised trial

Corresponding author:

Dr Jane Derges, School of Life Sciences, University of Westminster, 115 New Cavendish Street, London W1W 6UW

dergesj@westminster.ac.uk

Phone: 020 7911 5000 ext. 3884

Fax: 020 7911 5087

Co-authors:

Prof. Angela Clow, Psychology Department, University of Westminster, London, UK

a.clow@westminster.ac.uk

Ms Rebecca Lynch, School of Life Sciences, University of Westminster, London, UK

rebecca.j.lynch@googlemail.com

Dr Sumeet Jain, School of Life Sciences, University of Westminster, London, UK

sumeet.jain@yahoo.com

Dr Gemma Phillips, Institute for Health and Human Development, University of East London, UK; Gemma.phillips@lshtm.ac.uk

Prof. Mark Petticrew, Department of Public Health, London School of Hygiene and Tropical Medicine, London, UK; Mark.Petticrew@lshtm.ac.uk

Prof. Adrian Renton, Institute for Health and Human Development, University of East London, UK; adrian2@UEL-Exchange.uel.ac.uk

Dr Alizon Draper, School of Life Sciences, University of Westminster, London, UK

a.draper@westminster.ac.uk

Key words: Public health, programme intervention evaluation, qualitative study, interviews

For peer review only

ABSTRACT

Background
Well London is a multi-component community engagement and co-production programme designed to improve the health of Londoners living in socio-economically deprived neighbourhoods. To evaluate outcomes of the *Well London* interventions, a cluster randomized trial (CRT) was conducted that included a longitudinal qualitative component, which is reported here. The aim is to explore in depth the nature of the benefits to residents and the processes by which these were achieved.

Methods
The one-year longitudinal qualitative study was nested within the CRT. Purposive sampling was used to select three intervention neighbourhoods in London and 61 individuals within these neighbourhoods. The interventions comprised activities focused on: healthy eating (HE), physical exercise (PE) and mental health and well-being (MHWB). Interviews were conducted at inception and following completion of the *Well London* interventions to establish both if and how they had participated. Transcripts of the interviews were coded and analysed using Nvivo.

Results
Positive benefits relating to the formal outcomes of the CRT were reported, but only among those who participated in project activities. The extent of benefits experienced was influenced by factors relating to the physical and social characteristics of each neighbourhood. The highest levels of change occurred in the presence of: a) social cohesion, pre-existing but also as facilitated by *Well London* activities; b) personal and collective agency; c) involvement and support of external organisations. Where the physical and social environment remained unchanged, there was less participation and fewer benefits.

Conclusion
These findings show interaction between participation, well-being and agency, social interactions and cohesion, and that this modulated any benefits described. Pathways to change were thus complex and variable, but both personal well-being and local social cohesion emerged as important mediators of change.

Article summary: strengths and limitations of the study

Strengths
- Uses participants perspectives to identify why and how people participate in community health programmes
- Focuses on agency and its relationship to 'well-being' within an urban marginalised population
- Highlights the importance of social cohesion to 'well-being' and subsequently, participation in community health programmes

Limitations
- Further exploration of non-participation is required for future study
- An ethnographic focus would contribute both methodologically and to analysis

For peer review only

BACKGROUND

Processes of change in community-based interventions

Improving the health and well-being of populations living in disadvantaged areas of the UK remains a key public health challenge[1,2]. But while there is extensive evidence documenting the consequences and causes of health inequalities, less is known about the interplay between specific causal factors or what interventions are effective in reducing them[3]. There is growing recognition of the need to understand which interventions are effective and the processes or pathways by which effects are achieved[4-7]. This is particularly important for interventions that are “complex”[8,9] and in which local contextual factors modulate both the process of implementation and generation of outcomes[10]. The *Well London* programme is a complex intervention comprising multiple components and using a community engagement model. The term ‘participation’ is used here to highlight participants’ agency in relation to choice and whether or not they decided to take part in the interventions. The interventions comprised a series of activities based around healthy eating, physical activity and mental well-being. ‘Well-being’ is here defined as a eudemonic state in which the individual experiences positive attachment, a sense of meaningfulness and usefulness in life. This framework was used in each area but the delivery method varied according to local needs and priorities, as outlined in current theories concerning the design and evaluation of complex interventions[11,12]. Further details are obtainable from the *Well London* Website[13].

As Draper at al. and others note there is a need for rigorous evaluations that explore the causal pathways by which community participation influences health outcomes[14-16]. Popay[17] and Wallerstein[18] have hypothesized a number of possible pathways, but these remain largely unexamined. The relationship between social context, individual agency and participation has also been neglected[19], as well as exploration of the effects of interventions to address health inequalities[20] and the nature of personal agency and its relationship to a social determinants of health framework[21]. Whilst the social and environmental context in which people live and their ability to exercise individual agency in relation to decision-making about health is recognized as important, there are few qualitative studies that examine how these are interlinked and impact community engagement programmes.

The analysis presented here focuses on a qualitative study, which was embedded within the cluster randomized trial (CRT) of the *Well London* programme (see[22] for a full description of the trial protocol). The primary aim of the qualitative study was to examine the causal pathways that generated any intervention effects from the perspectives of local residents, who were involved as strategic partners in *Well London*’s design and delivery.

METHODS

Study Objectives

The objectives of the qualitative study were:

1. To identify how and why individuals participated in *Well London* activities;
2. To explore the different project components that enabled people to improve their health practices and sense of ‘well-being’;
3. To identify factors in the social and physical environment that influenced attitudes to health.

Study design

A longitudinal qualitative research element was included in the CRCT in order 1) to address the complexity[23] of the intervention; 2) to try to identify specific factors which enable or

For peer review only

obstruct individuals in leading healthy lives; 3) to understand subjective experience and the role of ‘agency’ in relation to participation.

The longitudinal qualitative study component was nested within the larger *Well London* CRT (for details of overall trial design, see[24]). It comprised a series of in depth interviews conducted over a period of one year, with interviews undertaken in two stages: firstly, at the implementation stage of the interventions and secondly, post-intervention. Both participants and non-participants of *Well London* were interviewed to capture whether exposure to the interventions would lead to neighbourhood level improvements in health and health practices, or whether direct participation was required. Limited observation of three selected *Well London* intervention neighbourhoods was also undertaken, to contextualise the interview data. Interviews were inductively examined in accordance with increasing calls for better capture of participants views[25].

Selection of study neighbourhoods

Initial observation showed contextual variation between the twenty *Well London* intervention neighbourhoods in environment, demography and history as well as in intensity and range of community activities running parallel to (i.e not commissioned by) *Well London*. A critical case sampling approach was therefore used to select 3 neighbourhoods to be included in the qualitative study (chosen in consideration of the in depth nature of the study). This approach selects cases based on criteria that are seen to be particularly important for the research project: “if it happens there, it will happen everywhere” or “if it doesn’t happen there it won’t happen anywhere”, “if that group is having problems we can be sure that every group is having problems”[26].

In selecting this approach it was necessary to identify what would make a critical case in relation to the objectives of the qualitative study. We therefore included neighbourhoods with both low and high levels of community projects. The method of programme delivery within *Well London* also differed and the three neighbourhoods chosen reflect this by including neighbourhoods with high and low levels of pre-existing community activities beyond those provided by the *Well London* programme, and differences in the manner of their delivery.

The first of these neighbourhoods (Eastford[1]) had a wide range of community activities offered prior to *Well London*, and continues to offer many activities unconnected to *Well London*. The second neighbourhood (Hartfield) has a core group of volunteers instrumental in generating engagement in *Well London* activities and they live in an enclosed geographical space (a housing estate). The third neighbourhood (Mountside) had limited community activities prior to *Well London* and a population dispersed among a number of differing housing sites. The manner of *Well London* delivery in these neighbourhoods varied from highly pro-active and involved members to a less cohesive and active method of delivery.

Study population

61 individuals were recruited at the start of *Well London* delivery and comprised matched participants of the interventions, and non-participants (see Table 1). Participants were purposively selected from within the interventions across the 3 study neighbourhoods and non-participants were selected through snowball sampling; these contacts were made by the researcher during visits to the neighbourhood. Selection for the qualitative study was based on providing theoretical insights rather than broader generalisations, as noted by Gardner and Chapple[27]. ‘Participant’ is here defined as residents who both received the *Well London* activities, and volunteered in their delivery.

---

[1] Pseudonyms are used throughout for places and people

For the post-intervention interview, a total of 45 agreed to a second interview. Reasons given by the 16 who did not attend this second interview were as follows: moved out of the neighbourhood (2), refusal of a follow-up interview (3), no response elicited (9), and illness (2). New recruits were not sought as changes over the intervening period would not have been captured.

Ethnicity, age and length of time in the neighbourhood among the study population were mixed across all three neighbourhoods; each neighbourhood showed variation according to all these categories, most noticeably, ethnicity. It is beyond the scope of this paper to examine the effects of this in detail, other than to recognize this as a difference requiring further investigation.

Table 1. Participant and non-participant profiles

1st Round interviews	Age range	Ethnicity	Gender	*Well London* participation	2nd Round interviews
Hartfield	16-25: 3<br>26-35: 8<br>36-45: 3<br>46-55: 5<br>56-65: 2<br>66-89: 1	African 13, Indian 3, Bangladeshi 1, White British 3, European (Lithuania) 1	Female: 16<br>Male: 5	Participants: 13<br>Non-participant: 8<br><br>Total: 21	Stage 2.<br>P=11<br>NP=8<br><br>Total: 20
Eastford	16-25: 4<br>26-35: 4<br>36-45: 2<br>46-55: 4<br>56-65: 3<br>66-75: 3	Bangladeshi 5, Indian 1, Pakistani 1, Caribbean 2, Black British 1, African 3, Chinese 2, White British 3, Irish 2	Female: 18<br>Male: 2	Participants: 11<br>Non-participants: 9<br><br>Total: 20	Stage 2.<br>P=7<br>NP=8<br><br>Total: 15
Mountside	16-25: 5<br>26-35: 3<br>36-45: 4<br>46-55: 3<br>56-65: 3<br>66-75: 2	White British 5, British Asian 5, Caribbean 4, European (Turkish) 3, African 2, Chinese 1	Female: 11<br>Male: 9	Participants: 10<br>Non-participant: 10<br><br>Total: 20	Stage 2.<br>P=6<br>NP=4<br><br>Total: 10

Data collection and analysis

Pre- and post intervention interviews used the same topic guide and focused on participants experiences of the *Well London* interventions and any reported changes to eating, exercise and mental health practices (see supplementary file). Also both participants and non-participants were asked for their views about the neighbourhood environment. Interviews were recorded and transcribed before being entered into Nvivo. Each transcript was checked for quality, coded and analysed using a framework based on Spencer, Ritchie and O'Conner's 'analytic hierarchy'[28]). This allowed systematic analysis of the large dataset but was flexible enough to allow refinements to the coding. Codes from interviews were identified and grouped under categories generated from the interview topics. Data were analysed not only deductively from the primary outcome measures (changes to healthy eating, physical activity and mental well-being), but also inductively from emerging themes identified from the interviews.

Observational data of the neighbourhoods was recorded both in photographs and written notes and included the local geography, amenities and range of community facilities and activities that were available. A separate researcher was employed at each stage of the study;

For peer review only - http://bmjopen.bmj.com/site/about/guidelines.xhtml

one researcher for stage 1 interviews, and the second researcher for stage 2. They each also conducted the observations simultaneous to the interviews.

Interview quality assurance and ethics

Quality assurance procedures were undertaken to minimise researcher bias when coding the interviews by randomly selecting three interviews, which were then recoded by two independent researchers blind to the initial coding. The three interviews were compared to identify new codes and establish a degree of consensus in applying a particular code to similar text. The University of Westminster Research Ethics Committee approved the study. Following initial telephone/email contact, written consent was obtained from every participant/non-participant. Each individual also received a short pamphlet describing the *Well London* project and an explanation of the qualitative component. Verbal explanation was also provided at the start of the interview process.

The results of the qualitative study are presented here by area. The reason for this is that the context and environment in which participants and non-participants were living and into which the *Well London* interventions and activities were introduced, has been shown to be a key factor in showing why individuals participated. Subsequently, data are presented by area, not theme.

RESULTS

Findings from the qualitative data show participants describing positive changes, both to their individual health and experiences of their neighbourhood as a result of participation in the *Well London* activities. However, equally significant was the degree of variation in how these changes were perceived between each neighbourhood, which was modulated by: mode of delivery, characteristics of individuals, neighbourhood history and attitudes to social interaction. As a consequence, each area is described separately in the results and the basis for using quotes is to represent what was said in relation to the themes.

Overall, participants identified the importance of social interaction as a crucial component of participation in the *Well London* activities. For example: a social gathering that included eating healthy food; gardening and opportunities to chat over a cup of tea; women feeling safer when sharing an evening walk together. Participation in practical health-related activities were only beneficial within a social context. By comparison, non-participants, despite their individual attempts at improving their own health, experienced no benefits either from efforts to change eating or physical activity levels, or from being around others in the community who were participating in *Well London* activities:

> *'So I was left a very lonely bunny for quite a while. I don't like going out walking all the time on my own, I don't like going swimming on my own. I love to do it, but I don't like doing it on my own. If there was a group going, I would go'* Mary, age 48, Irish, non-participant

Furthermore, a small number of non-participants felt excluded from the *Well London* interventions, suggesting there may be some non-beneficial effects. For example, in response to a question about positive changes on the estate as a result of *Well London*, one resident commented:

> *'Well, that "getting better" is a matter of opinion, because I look on it now as a ghetto. It was an unruly estate before. It has quietened down, but now it's a ghetto. I don't go out anymore, I don't do anything anymore. No. No. And I hardly even*

For peer review only - http://bmjopen.bmj.com/site/about/guidelines.xhtml

> *talk to people now. I mean, I'll sit out at my doorstep and, you know, a lot of people'll stop and chat to me, but I don't really like it'.* Karen, age 54, White British, non-participant

Despite scoring high on the 'Indices of Deprivation'[29], the three *Well London* intervention neighbourhoods will be described separately in acknowledgement of their diversity and to bring out the nuances of how place impacts participation and any consequent outcomes.

HARTFIELD is a large housing estate built in the 1950s, comprising low-rise blocks constructed around a series of rectangular grassed areas. Although the most homogenous of the three neighbourhoods in terms of population and environment, prior to *Well London* it was socially fragmented with a dearth of community activities.
Pre-intervention descriptions of Hartfield included:

> *'And the word 'Hartfield' put horror – it was notorious. Everybody who was difficult was dumped here'.* Margaret, 59, White British

> *It's a dumping ground. It always has been a dumping ground. You know? I begged not to be put on there. I've been there 21 years.* Liz, 48, Irish

> *I will tell you straightaway there was no life in the community before the arrival of* Well London*. No, that is the summary of the whole thing; where you are living in an area where there was no life.* Clifford, 46, African (Uganda)

Post-intervention, Hartfield respondents reported the most substantive change in experienced health benefits of all 3 neighbourhoods. Factors that facilitated this included: a) a pro-active, charismatic Well-London coordinator; b) increased safety following changes in policing methods on the estate, instigated by the coordinator; c) a high number of proactive volunteers; and d) residents as stakeholders through the estate's Residents Committee, set up by *Well London*. Benefits described included: enhanced feeling of social cohesion, new knowledge about health, involvement in estate-wide activities, improved relations with neighbours, less complaints about the neighbourhood's lack of cleanliness, safety and violence.

Box 1 Hartfield	Reported benefits in HE, PA, MHWB, and Social Interaction
**Healthy Eating**	➢ *So you know, after walk we have this exercise to stretch ourselves, and then after that we used to have fruit. Yeah, so we used to sit in the park and we used to eat fruit and that's how I learn to eat fruit basically.* Priya, 34, Indian<br>➢ *Oh my God, people are healthier now. It's changed, it's completely changed. I say that it's changed because I am involved - I know how much to my own particular health* (and*) the health of my family and how much has changed. I'm able to know more now, I know what to eat, what not to eat.* Thomas, 45, African (Ghana)

For peer review only - http://bmjopen.bmj.com/site/about/guidelines.xhtml

**Physical Activity**	➢ *Like before, you know, I used to find walking was kind of one of the painful things, yeah, I wouldn't bother to walk, I would rather take bus rather than walking, but now I feel like, now - rather than taking bus or anything, let's just walk, it's not going to take me that long.* Sandra, 43, African (Uganda)<br>➢ *And we have some people who want to go night walk - like the Somalians. If the place is dark, they would like to walk. Because of, you know, night-time you can also wear your trousers - so that they can walk faster.* Joyce, 38, African (Nigeria)
**Mental Health, Wellbeing**	➢ *The fact that it's made me proud of myself and the whole project and the whole community, because it's made people come in to do the activities.* Bernard 42, African (Ivory Coast)<br>➢ *It has made the community come together, that's what I've seen anyway, people have come together, which is very good.* Claudette, 37, African (Sierra Leone)
**Social interaction**	➢ *Yes we used to be on our own, nobody say hello to each other, but because of Cheryl,* Well London *came to this place* (and*) it start connecting us.* Lorraine, 39, African (Uganda)<br>➢ *Yeah the police, which are responsible to this area, yes it's changed a lot because now they can say 'hello' to you. Sometimes even the kids, if they see them playing outside they will stand and speak with them, and ask them 'are you with elderly adults or are you alone?' And so forth and we are happy for that.* Margaret, 41, African (Ghana)<br>➢ *I think that it does a good thing -* Well London *came to help out. We did a food basket with five foods, I did that as well so. I know the women come to do, they have sewing classes, and it's just - but it's for the coming together, the community together, that's what I think.* Clara, 42, Bangladeshi<br>➢ *I've been proud to say that this is one of my proudest periods in regards to this community. Yeah, this is because our efforts that has been put in place by* Well London *and followed by* Well London *volunteers. The Hartfield estate now compared to what it was in the past, it's a name at least to be proud of.* Frank, 39, African (Nigerian)

EASTFORD has undergone extensive regeneration over the last decade, including funding to develop community projects that promote health. This had generated an ethos of community participation and differentiating the *Well London* interventions from these other activities was subsequently more difficult, especially its effects on Mental Health and Well-being. Despite this, the positive changes experienced here by participants refer specifically to the *Well London* activities.

Pre-intervention, Eastford was already defined as a place where things happened: 'Eastford *is great – there's so much to do here!'* Jermina, 26, Bangladeshi.
Post-intervention, respondents experienced some change. Benefits included a) a sense of autonomy from volunteering and involvement in managing and running activities; b) feeling

productive and useful; c) increased knowledge of food/cooking & improved health; and c) enhanced feeling of social cohesion.

Box 2 Eastford	Reported benefits in HE, PA, and MHWB and Social Interaction
**Healthy Eating**	➢ *Yeah, whereas say somebody comes in on Tuesday and does a little bit of cooking - it's quite quick, but with the 'Cook & Eat' it was more in depth and they explained things better and you could ask questions and all things like that, yeah, it was much better.* Clare, 38, White British<br>➢ *Earlier I used to be like, junkie foods eating; crisps and all those things. Now it's like more fruit and vegetables and salad in my diet.* Shubha, 28, Indian
**Physical Activity**	➢ *It was great, it's fantastic - I cannot express how good it is to get in there and get your hands dirty, and to see everybody else doing the same thing.* Sarah, 34, White British<br>➢ *Yes, I do a lot, because I'm doing them exercises it's helped me, it's good for my health, I feel much better, I can breathe properly. And you make friend. Yeah, it's good for me - I go out, and you meet friends.* Tricia, 72, Caribbean
**Mental Health and Well-being**	➢ *I feel I can keep my mind going and I feel like my mind has to be active because I don't want to sit down and get depressed or something. If I think bad things then I won't be doing nothing and I don't want to go like that yeah.* Maureen, 48, Irish
**Social interaction**	➢ *I feel so much more confident that we can make this move on; the thing we were given was confidence building. I think that sort of confidence building was something I didn't see – yeah, running an organisation, running that level of budgeting and planning.* Michael, 50, White British<br>➢ *You can see it, just a healthier lifestyle: people busy all the time, people - not so much arguments and you see that less and people are a lot more sociable as well.* Pat, 36, Black British

MOUNTSIDE

Mountside is a neighbourhood of contradictions, characterized by a geographically dispersed, ethnically and socio-economically diverse but transitory population and a reverse trend in terms of regeneration:

> *'It was a transient population so you'd get people move in for three months, as I say, trash the place or do whatever'*. Paula, 45, White British

> *'It became what I can only describe as a dumping ground for literally anybody. There was no perspective on who was living where and next to whom; people were just thrown into the flats regardless of background, criminal intention or anything'.* Mohan, 52, Indian

'When we first moved here it was gorgeous. Oh, you couldn't have wished for a more idyllic place to live. It was quiet, it was flowers, it was lovely neighbours. But of course, a lot of our neighbours then had been here since the block went first up in the '60s, so they were all getting old and consequently all started to die and then their families sold the flats to housing associations. And you go from there'. Monica, 62, White British

The loss of facilities such as a local cinema, shops and other community activities resulted in the main street consisting of fast food outlets and budget shops – high street brands that used to exist had moved away, apart from a large supermarket. There were pockets of privately owned terraced housing divided from local authority tower blocks, marking a clear socio-economic boundary. Attitudes to *Well London* were similarly divided; some viewed the interventions positively, and some not.

Post-intervention, Mountside respondents recounted little change; positive change was commented on only in relation to the Mental Health and Well-being activities. Factors that prevented change were: a) lack of effective, coordinated local leadership; b) dispersal and transience of the local population; c) lack of cohesive environmental planning; and d) strong sense of neglect and 'being forgotten' by residents.

Box 3 Mountside	Reported benefits in HE, PA, and MHWB
**Healthy Eating**	➢ *Yeah, you know children like chips, sausages, yeah. Just sometimes I'm cooking chips - every time Turkish foods; rice yeah. You know, my older one all the time she wants outside, McDonalds, chicken, chips, she's eating too much. And everywhere this food. I'm telling her 'you know, too much oily inside, you no eat' and she's not listening to me.* Hanife, 36, Turkish<br>➢ *You can see the higher fast food intake, zero exercise, high alcohol and stressful kind of lifestyles that people lead. And this is also supported by the number of fast food outlets that thrive in these areas.* Mohan, 31, Asian British
**Physical Activity**	➢ *We've got one park over the road but, again that's a dangerous place. We've had murders over there, we've had people killing the swans to eat and people sleeping rough over there. So of course, parents weren't taking their kids over to the park, and you can't blame them, I wouldn't go over there.* Marie, 46, White British
**Mental Health and Well-being**	➢ *It was fantastic, it brought up a lot of issues and a lot of practices and things that I'm already aware of, and I really, really enjoyed it'.* Saroja, 27, Asian British<br>➢ *Oh yeah, and I wish it could continue, I really, really do, because I think it's started to actually break down a few barriers. We were all really sad when it ended and I thought; this is something that could really build up. And I just wish we could have* Well London *permanently. It was a really nice thing, and because it came to this area it made us think, well we are important, it's come here. I know it came here because we were a deprived area but people are listening to us.*

	They're trying to do something to help us. And like I say, the worst thing is that we haven't got it (now). *If you can bring it back I'd be ever so grateful and so would a lot of other people.* Karen, 41, White British<br>➢ *I came away having learnt a lot more about the other women – appreciating them more, yeah, I think that's word should be put in there; appreciating other people, not just cultures but people themselves*. Molly, 45, Caribbean
**Social interaction**	➢ *It takes the form of exercise when I can be bothered. I will say I'm a bit lazy sometimes, so you'll do it and then it's like you don't want to take it on, on your own, so you do need motivation* Jan, age 36, White British

CONCLUSION

Participants described an overall positive impact from the *Well London* project activities, but the data also reveal a complex and nuanced picture of if and how outcomes were achieved with two key findings. Firstly, it shows how neighbourhood-level changes did not lead to benefits amongst those who did not participate in project activities. Secondly, the characteristics of neighbourhoods, both social and physical, were fundamental in moderating whether people participated, the nature and extent of the consequent benefits, and any reported changes in health practices. Therefore, participation is dependent on the provision of particular elements that support it; namely a socially cohesive environment in which to get to know neighbours; a safe environment that is well-maintained; access to affordable, nutritious food; a degree of autonomy that allows residents to be involved in decision-making and thereby improve confidence and self-esteem.

The role of the *Well London* co-ordinators also emerged as an important theme across the three areas. With their active involvement through co-ordinated organisation of volunteers, a commitment to the area shown by their understanding of local issues, participation was more successfully implemented. In Hartfield for example, residents frequently cited their co-ordinator; as a 'boundary crosser'[30], she was pivotal in encouraging and facilitating their involvement in activities. As a she By contrast, co-ordination of the activities in Mountside were deemed problematic by many - apart from those attending DIY Happiness groups, which were identified as positive because they acknowledged residents' sense of deprivation. Activities that did less well were those deemed to be out of touch with local needs i.e 'fun' activities were less successful than those seen to have direct relevance and benefit, such as stress management. In Eastford, residents were encouraged to lead projects and be involved in decision-making and subsequently, external *Well London* leadership and co-ordination was mentioned less, whereas the benefits of taking a leadership role were spoken of frequently.

Well-being was a central requirement for the exercise of personal agency, which in turn enabled participation in the *Well London* activities. Well-being was tied to factors such as being able to live in a socially cohesive and safe neighbourhood where neighbours respected one another and where problems were recognised and acted upon by local authorities. Once engaged there was an apparent feedback loop whereby further enhancement of well-being increased personal agency and lead to increased involvement in the activities which then lead to changes in attitudes and practices in eating, exercise and mental health. Participants' well-being, agency and participation also interacted with their sense of place, again in an iterative fashion. Following improvements to the physical environment, such as direct involvement of

For peer review only - http://bmjopen.bmj.com/site/about/guidelines.xhtml

local police in providing safer spaces, further enhancement of well-being and agency were described. Well-being in this instance, appears to be a crucial mediator between agency, participation and improved health practices. A recent review of individual experiences of community engagement also found that active participation in community initiatives has important psychosocial benefits for participants that include enhanced feelings of personal confidence and self-esteem, as well as enhanced social relationships and social cohesion within a community[31, 32].

In this study, participation was not a simple binary variable and quantitative measures alone did not pick up the subtleties and complex variations. Our findings show that participation is a complex and dynamic process with well-being at its core, acting as a catalyst that enables participation through a related sense of personal agency. Through further enhancement of well-being and associated social cohesion, improvements in health practices were experienced, just as in its absence no benefits were recounted. However, participation was not universally desired; some reported feeling excluded from the *Well London* interventions and the subsequent changes taking place in the neighbourhood (see participant comment, pg 6), whilst others reported little interest in taking part because improving health was neither a priority nor an personal goal.
These findings also confirm that health practices are not a separate 'capsule' of behaviour[33, 34], but are embedded within particular social, cultural and physical milieus. Across the three neighbourhoods however, there was a clear gradient of change with the greatest change seen in the presence of; a) involvement and support of external organisations; b) personal and collective agency enhanced by effective leadership in project activities; c) social cohesion fostered by *Well London* activities. Where the physical and social environment remained unchanged, there was less participation and therefore fewer benefits. Also, each area reflected considerable variation in levels of maturity and self-management: each was graded in terms of what progress was possible, Mountside being at the beginning of area level change with safety and environmental pollution still an issue in contrast to Eastford, which featured a more developed and progressive attitude due to investment both financial and from the local authority. Hartfield was in the midst of significant degrees of change through both necessity and a relatively recent influx of enthusiastic residents supported by an equally enthusiastic co-ordinator.

As others have identified, the dynamics of participation from the perspective of individual agency have been neglected[17, 18, 32]. In addressing this, our findings show participation as a dynamic and flexible process with agency at its core. Also, the community engagement approach fed back into and reinforced feelings of well-being and agency and thus encouraged and supported changes in health practices. The findings are consistent with elements of Popay's[17] proposed pathways by which community engagement leads to health outcomes, and specifically that social capital/cohesion and community empowerment are important intermediaries[17]. Additionally they point to the need for further understanding of how these interact with agency, well-being and empowerment at the individual level and in different social contexts, in order to achieve inclusive engagement with such programmes. As Popay argues[25], people's own ideas need to be incorporated fully in the design and delivery of proposed health interventions.

For peer review only

Acknowledgements We thank all of the study participants for giving generously of their time; the project delivery partners for their assistance; our colleagues at the University of East London, University of Westminster and the London School of Hygiene and Tropical Medicine for their support and cooperation.

Author contributions: J Derges data collection, interpretation, analysis and writing; S Jain, data interpretation and analysis; A Clow, data analysis and interpretation; R Lynch, data collection, data analysis; A Renton, project lead; A Draper, data interpretation, analysis and writing.

Funding: This work was supported by the Wellcome Trust, Grant code: 083679/Z/07/Z

Competing interests: None

Ethics approval: granted by University of Westminster Ethics Committee

Data sharing statement: Anonymised interview transcripts may be obtained on request from Dr A. Draper, University of Westminster

For peer review only

REFERENCES

1. WHO: **The Jakarta Declaration on Leading Health Promotion into the 21st Century**. Fourth International Conference on Health Promotion, Jakarta, 1997.
2. Marmot M: **The Marmot Review: Fair Society, Healthy lives – Strategic Review of Health Inequalities in England post-2010**. London, 2010.
3. Whitehead M: **A typology of actions to tackle social inequalities in health**. Journal of Epidemiology and Community Health 2007;**61:**473-478.
4. Anderson A: **The Community Builder's Approach to Theory of Change: A Practical Guide to Theory Development**. New York: The Aspen Institute Roundtable on Community Change 2005.
5. Bryce J, Victora CG, Habicht JP, Black RE, et al. (MCE-IMCI Technical Advisors): **Programmatic pathways to child survival: results of a multi-country evaluation of Integrated Management of Childhood Illness**. Health Policy and Planning Suppl 2005;**1:**15-117.
6. O'Dwyer LA, Baum F, Kavanagh A, MacDougall C: **Do area-based interventions to reduce health inequalities work? A systematic review of evidence**. Critical Public Health, 2007;**17**:317-335.
7. Victora CG, Habicht J-P, Bryce J: **Evidence-based public health: moving beyond randomized trials.** American Journal of Public Health 2004;**94**:400-405.
8. Medical Research Council: **A framework for development and evaluation of RCTs for complex interventions to improve health.** London: MRC; 2000.
9. Medical Research Council: **Developing and evaluating complex interventions: new guidance.** London: MRC; 2008.

For peer review only

10. Hawe P , Sheill A, Riley T, Gold, L: **Methods for exploring implementation variation and local context within a cluster randomized community intervention trial**. Journal of Epidemology and Community Health 2004;**58**:788-793.

11. Ogilvie D, Cummins S Petticrew M, White M, Jones A, Wheeler K: **Assessing the evaluability of complex public health interventions: five questions for researchers, funders, and policymakers.** Milbank Q 2011,**89**:206-225.

12. Hawe P, Shiell A, Riley T: **Complex interventions: how "out of control" can a randomized controlled trial be?** BMJ 2004, **328**: 1561-1563

13. **Well London: communities working together for a healthier city**. [http://www.welllondon.org.uk]

14. Draper AK, Hewitt G, & Rifkin SB: **Chasing the Dragon: developing indicators for the assessment of community participation in health programmes**. Soc Sci Med 2010,71:1102-1109. DOI 10.1016/j.socscimed.2010.05.016

15. Milton B, Attree P, French B, Povall S, Whitehead M, Popay J: **The impact of community engagement on health and social outcomes: a systematic review**. Community Development Journal 2012;**47**(3): 316-334.

16. Preston R, Waugh H, Larkins S, Taylor J: **Community participation in rural primary health care: intervention or approach?** Australian Journal of Primary HealthCare 2010;**16**:4-16.

17. Popay J: **Community engagement and community development and health improvement: a background paper for NICE**. London: NICE 2006.

18. Wallerstein N: **What is the evidence on effectiveness of empowerment to improve health?** Copenhagen, Denmark, WHO Regional Office for Europe, 2006. Health Evidence Network report; http://www.euro.who.int/Document/E88086.pdf. (accessed January 2013)

For peer review only

19. Cleaver F: **Institutions, agency and the limitations of participatory approaches to development**. In: Hickey S, Mohan G, eds. Participation: From Tyranny to Transformation? London Zed Books, 2004:271-283.

20. Egan M, Tannahill C, Petticrew M, Thomas, S: **Psychosocial risk factors in home and community settings and their associations with population health and health inequalities: a systematic meta-review.** BMC Public Health 2008;**8**:239

21. Forde I, Raine R: **Placing the individual within a social determinants approach to health inequity**. The Lancet 2008;**122**(11):1177-1187.

22. Wall M, Hayes R, Moore D, Petticrew M, Clow A, Schmidt E, Draper A, Lock K, Lynch R, Renton A: **Evaluation of community level interventions to address social and structural determinants of health: a cluster randomised controlled trial**. BMC Public Health 2009;9:207 DOI:10.1186/1471-2458-9-207.

23. Petticrew M: **When are complex interventions 'complex'? When are simple interventions 'simple'?** Euro J Public health 2011;**21**:397-8.

24. Phillips G, Renton A, Moore DG, Bottomley C, Schmidt E, Lais S, Yu G, Wall M, Tobi P, Frostick C, Clow A, Lock K, Petticrew, Hayes R: **The Well London program – a cluster randomized trial of community engagement for improving health behaviors and mental well-being: baseline survey results.** Trials 2012 Jul 6;13:105.doi: 10.1186/1745-6215-13-195.

25. Popay J: **Whose theory is it anyway?** Journal of Epidemiology and Community Medicine 2006;**60**:571-572.

26. Patton MQ: **Qualitative Research and Evaluation Methods**. 3rd Edition. Sage Publications, Thousand Oaks, California 2002:236.

27. Gardner K, Chapple A: **Barriers to referral in patients with angina: a qualitative study**. BMJ 1999;319-321.

For peer review only

28. Spencer L, Ritchie J, O'Connor W: **Analysis: Practices, principles and processes**. In *Qualitative research practice: a guide for social science students and researchers.* Edited by J. Ritchie & J Lewis. Thousand Oaks, CA: Sage 2003:200-218.

29. **English indices of deprivation 2010 Government Report** http://www.communities.gov.uk/publications/corporate/statistics/indices2010

30. Clow, A Hamer, M: **The iceberg of social disadvantage and chronic stress: implications for public health.** Neurosci Biobehav Rev 2010;**35**:1.

31. Attree P, French B, Milton B, Povall S, Whitehead M, Popay J: **The experience of community engagement for individuals: a rapid review of the evidence**. Health and Social Care in the Community 2011;**19**:250-60.

32. Polgar S. **Health and human behaviour: areas of interest common to the social and medical social sciences**. Current Anthropology 1962;**3**:159-205

33. Fienieg B, Nierkins V, Tonkens E, Plochg T, Stronks K. **Why play an active role? A qualitative examination of lay citizens' main motives for participation in health promotion**. Health Promotion International 2012;**27**:416-26.

34. Kilpatrick S, Cheers B, Gilles M, Taylor J: **Boundary crossers, communities, and health: exploring the role of rural health professionals**. Health Place 2009;**15**:284-290

For peer review only

‘*Well London*’ and the benefits of participation: results of a qualitative study nested in a cluster randomised trial

Corresponding author:

Dr Jane Derges, School of Life Sciences, University of Westminster, 115 New Cavendish Street, London W1W 6UW

dergesj@westminster.ac.uk

Phone: 020 7911 5000 ext. 3884

Fax: 020 7911 5087

Co-authors:

Prof. Angela Clow, Psychology Department, University of Westminster, London, UK

a.clow@westminster.ac.uk

Ms Rebecca Lynch, School of Life Sciences, University of Westminster, London, UK

rebecca.j.lynch@googlemail.com

Dr Sumeet Jain, School of Life Sciences, University of Westminster, London, UK

sumeet.jain@yahoo.com

Dr Gemma Phillips, Institute for Health and Human Development, University of East London, UK; Gemma.phillips@lshtm.ac.uk

Prof. Mark Petticrew, Department of Public Health, London School of Hygiene and Tropical Medicine, London, UK; Mark.Petticrew@lshtm.ac.uk

Prof. Adrian Renton, Institute for Health and Human Development, University of East London, UK; adrian2@UEL-Exchange.uel.ac.uk

Dr Alizon Draper, School of Life Sciences, University of Westminster, London, UK

a.draper@westminster.ac.uk

For peer review only

For peer review only - http://bmjopen.bmj.com/site/about/guidelines.xhtml

ABSTRACT

Background
Well London is a multi-component community engagement and co-production programme designed to improve the health of Londoners living in socio-economically deprived neighbourhoods. To evaluate outcomes of the *Well London* interventions, a cluster randomized trial (CRT) was conducted that included a longitudinal qualitative component, which is reported here. The aim is to explore in depth the nature of the benefits to residents and the processes by which these were achieved.

Methods
The one-year longitudinal qualitative study was nested within the CRT. Purposive sampling was used to select three intervention neighbourhoods in London and 61 individuals within these neighbourhoods. The interventions comprised activities focused on: healthy eating (HE), physical exercise (PE) and mental health and well-being (MHWB). Interviews were conducted at inception and following completion of the *Well London* interventions to establish both if and how they had participated. Transcripts of the interviews were coded and analysed using Nvivo.

Results
Positive benefits relating to the formal outcomes of the CRT were reported, but only among those who participated in project activities. The extent of benefits experienced was influenced by factors relating to the physical and social characteristics of each neighbourhood. The highest levels of change occurred in the presence of: a) social cohesion, pre-existing but also as facilitated by *Well London* activities; b) personal and collective agency; c) involvement and support of external organisations. Where the physical and social environment remained unchanged, there was less participation and fewer benefits.

Conclusion
These findings show interaction between participation, well-being and agency, social interactions and cohesion, and that this modulated any benefits described. Pathways to change were thus complex and variable, but both personal well-being and local social cohesion emerged as important mediators of change.

Key words: Public health, programme intervention evaluation, qualitative study, interviews

Article summary: strengths and limitations of the study

Strengths

- Uses participants perspectives to identify why and how people participate in community health programmes
- Focuses on agency and its relationship to 'well-being' within an urban marginalised population
- Highlights the importance of social cohesion to 'well-being' and subsequently, participation in community health programmes

Limitations

- Further exploration of non-participation is required for future study
- An ethnographic focus would contribute both methodologically and to analysis

For peer review only - http://bmjopen.bmj.com/site/about/guidelines.xhtml

BACKGROUND

Processes of change in community-based interventions

Improving the health and well-being of populations living in disadvantaged areas of the UK remains a key public health challenge[1,2]. But while there is extensive evidence documenting the consequences and causes of health inequalities, less is known about the interplay between specific causal factors or what interventions are effective in reducing them[3]. There is growing recognition of the need to understand which interventions are effective and the processes or pathways by which effects are achieved[4-7]. This is particularly important for interventions that are "complex"[8,9] and in which local contextual factors modulate both the process of implementation and generation of outcomes[10]. The *Well London* programme is a complex intervention comprising multiple components and using a community engagement model. The term 'participation' is used here to highlight participants' agency in relation to choice and whether or not they decided to take part in the interventions. The interventions comprised a series of activities based around healthy eating, physical activity and mental well-being. 'Well-being' is here defined as a eudemonic state in which the individual experiences positive attachment, a sense of meaningfulness and usefulness in life. This framework was used in each area but the delivery method varied according to local needs and priorities, as outlined in current theories concerning the design and evaluation of complex interventions[11,12]. Further details are obtainable from the *Well London* Website[13].

As Draper at al. and others note there is a need for rigorous evaluations that explore the causal pathways by which community participation influences health outcomes[14-16]. Popay[17] and Wallerstein[18] have hypothesized a number of possible pathways, but these remain largely unexamined. The relationship between social context, individual agency and participation has also been neglected[19], as well as exploration of the effects of interventions to address health inequalities[20] and the nature of personal agency and its relationship to a social determinants of health framework[21]. Whilst the social and environmental context in which people live and their ability to exercise individual agency in relation to decision-making about health is recognized as important, there are few qualitative studies that examine how these are interlinked and impact community engagement programmes.

The analysis presented here focuses on a qualitative study, which was embedded within the cluster randomized trial (CRT) of the *Well London* programme (see[22] for a full description of the trial protocol). The primary aim of the qualitative study was to examine the causal pathways that generated any intervention effects from the perspectives of local residents, who were involved as strategic partners in *Well London*'s design and delivery.

METHODS

Study Objectives

The objectives of the qualitative study were:

1. To identify how and why individuals participated in *Well London* activities;
2. To explore the different project components that enabled people to improve their health practices and sense of 'well-being';
3. To identify factors in the social and physical environment that influenced attitudes to health.

Study design

A longitudinal qualitative research element was included in the CRCT in order 1) to address the complexity[23] of the intervention; 2) to try to identify specific factors which enable or

For peer review only - http://bmjopen.bmj.com/site/about/guidelines.xhtml

obstruct individuals in leading healthy lives; 3) to understand subjective experience and the role of 'agency' in relation to participation.

The longitudinal qualitative study component was nested within the larger *Well London* CRT (for details of overall trial design, see[24]). It comprised a series of in depth interviews conducted over a period of one year, with interviews undertaken in two stages: firstly, at the implementation stage of the interventions and secondly, post-intervention. Both participants and non-participants of *Well London* were interviewed to capture whether exposure to the interventions would lead to neighbourhood level improvements in health and health practices, or whether direct participation was required. Limited observation of three selected *Well London* intervention neighbourhoods was also undertaken, to contextualise the interview data. Interviews were inductively examined in accordance with increasing calls for better capture of participants views[25].

Selection of study neighbourhoods

Initial observation showed contextual variation between the twenty *Well London* intervention neighbourhoods in environment, demography and history as well as in intensity and range of community activities running parallel to (i.e not commissioned by) *Well London*. A critical case sampling approach was therefore used to select 3 neighbourhoods to be included in the qualitative study (chosen in consideration of the in depth nature of the study). This approach selects cases based on criteria that are seen to be particularly important for the research project: "if it happens there, it will happen everywhere" or "if it doesn't happen there it won't happen anywhere", "if that group is having problems we can be sure that every group is having problems"[26].

In selecting this approach it was necessary to identify what would make a critical case in relation to the objectives of the qualitative study. We therefore included neighbourhoods with both low and high levels of community projects. The method of programme delivery within *Well London* also differed and the three neighbourhoods chosen reflect this by including neighbourhoods with high and low levels of pre-existing community activities beyond those provided by the *Well London* programme, and differences in the manner of their delivery.

The first of these neighbourhoods (Eastford[1]) had a wide range of community activities offered prior to *Well London*, and continues to offer many activities unconnected to *Well London*. The second neighbourhood (Hartfield) has a core group of volunteers instrumental in generating engagement in *Well London* activities and they live in an enclosed geographical space (a housing estate). The third neighbourhood (Mountside) had limited community activities prior to *Well London* and a population dispersed among a number of differing housing sites. The manner of *Well London* delivery in these neighbourhoods varied from highly pro-active and involved members to a less cohesive and active method of delivery.

Study population

61 individuals were recruited at the start of *Well London* delivery and comprised matched participants of the interventions, and non-participants (see Table 1). Participants were purposively selected from within the interventions across the 3 study neighbourhoods and non-participants were selected through snowball sampling; these contacts were made by the researcher during visits to the neighbourhood. Selection for the qualitative study was based on providing theoretical insights rather than broader generalisations, as noted by Gardner and Chapple[27]. 'Participant' is here defined as residents who both received the *Well London* activities, and volunteered in their delivery.

---

[1] Pseudonyms are used throughout for places and people

For peer review only - http://bmjopen.bmj.com/site/about/guidelines.xhtml

For the post-intervention interview, a total of 45 agreed to a second interview. Reasons given by the 16 who did not attend this second interview were as follows: moved out of the neighbourhood (2), refusal of a follow-up interview (3), no response elicited (9), and illness (2). New recruits were not sought as changes over the intervening period would not have been captured.

Ethnicity, age and length of time in the neighbourhood among the study population were mixed across all three neighbourhoods; each neighbourhood showed variation according to all these categories, most noticeably, ethnicity. It is beyond the scope of this paper to examine the effects of this in detail, other than to recognize this as a difference requiring further investigation.

Table 1. Participant and non-participant profiles

1st Round interviews	Age range	Ethnicity	Gender	*Well London* participation	2nd Round interviews
Hartfield	16-25: 3<br>26-35: 8<br>36-45: 3<br>46-55: 5<br>56-65: 2<br>66-89: 1	African 13, Indian 3, Bangladeshi 1, White British 3, European (Lithuania) 1	Female: 16<br>Male: 5	Participants: 13<br>Non-participant: 8<br><br>Total: 21	Stage 2.<br>P=11<br>NP=8<br><br>Total: 20
Eastford	16-25: 4<br>26-35: 4<br>36-45: 2<br>46-55: 4<br>56-65: 3<br>66-75: 3	Bangladeshi 5, Indian 1, Pakistani 1, Caribbean 2, Black British 1, African 3, Chinese 2, White British 3, Irish 2	Female: 18<br>Male: 2	Participants: 11<br>Non-participants: 9<br><br>Total: 20	Stage 2.<br>P=7<br>NP=8<br><br>Total: 15
Mountside	16-25: 5<br>26-35: 3<br>36-45: 4<br>46-55: 3<br>56-65: 3<br>66-75: 2	White British 5, British Asian 5, Caribbean 4, European (Turkish) 3, African 2, Chinese 1	Female: 11<br>Male: 9	Participants: 10<br>Non-participant: 10<br><br>Total: 20	Stage 2.<br>P=6<br>NP=4<br><br>Total: 10

Data collection and analysis

Pre- and post intervention interviews used the same topic guide and focused on participants experiences of the *Well London* interventions and any reported changes to eating, exercise and mental health practices (see supplementary file). Also both participants and non-participants were asked for their views about the neighbourhood environment. Interviews were recorded and transcribed before being entered into Nvivo. Each transcript was checked for quality, coded and analysed using a framework based on Spencer, Ritchie and O'Conner's 'analytic hierarchy'[28]). This allowed systematic analysis of the large dataset but was flexible enough to allow refinements to the coding. Codes from interviews were identified and grouped under categories generated from the interview topics. Data were analysed not only deductively from the primary outcome measures (changes to healthy eating, physical activity and mental well-being), but also inductively from emerging themes identified from the interviews.

Observational data of the neighbourhoods was recorded both in photographs and written notes and included the local geography, amenities and range of community facilities and activities that were available. A separate researcher was employed at each stage of the study;

For peer review only - http://bmjopen.bmj.com/site/about/guidelines.xhtml

one researcher for stage 1 interviews, and the second researcher for stage 2. They each also conducted the observations simultaneous to the interviews.

Interview quality assurance and ethics

Quality assurance procedures were undertaken to minimise researcher bias when coding the interviews by randomly selecting three interviews, which were then recoded by two independent researchers blind to the initial coding. The three interviews were compared to identify new codes and establish a degree of consensus in applying a particular code to similar text. The University of Westminster Research Ethics Committee approved the study. Following initial telephone/email contact, written consent was obtained from every participant/non-participant. Each individual also received a short pamphlet describing the *Well London* project and an explanation of the qualitative component. Verbal explanation was also provided at the start of the interview process.

The results of the qualitative study are presented here by area. The reason for this is that the context and environment in which participants and non-participants were living and into which the *Well London* interventions and activities were introduced, has been shown to be a key factor in showing why individuals participated. Subsequently, data are presented by area, not theme.

RESULTS

Findings from the qualitative data show participants describing positive changes, both to their individual health and experiences of their neighbourhood as a result of participation in the *Well London* activities. However, equally significant was the degree of variation in how these changes were perceived between each neighbourhood, which was modulated by: mode of delivery, characteristics of individuals, neighbourhood history and attitudes to social interaction. As a consequence, each area is described separately in the results and the basis for using quotes is to represent what was said in relation to the themes.

Overall, participants identified the importance of social interaction as a crucial component of participation in the *Well London* activities. For example: a social gathering that included eating healthy food; gardening and opportunities to chat over a cup of tea; women feeling safer when sharing an evening walk together. Participation in practical health-related activities were only beneficial within a social context. By comparison, non-participants, despite their individual attempts at improving their own health, experienced no benefits either from efforts to change eating or physical activity levels, or from being around others in the community who were participating in *Well London* activities:

> *'So I was left a very lonely bunny for quite a while. I don't like going out walking all the time on my own, I don't like going swimming on my own. I love to do it, but I don't like doing it on my own. If there was a group going, I would go'* Mary, age 48, Irish, non-participant

Furthermore, a small number of non-participants felt excluded from the *Well London* interventions, suggesting there may be some non-beneficial effects. For example, in response to a question about positive changes on the estate as a result of *Well London*, one resident commented:

> *'Well, that "getting better" is a matter of opinion, because I look on it now as a ghetto. It was an unruly estate before. It has quietened down, but now it's a ghetto. I don't go out anymore, I don't do anything anymore. No. No. And I hardly even*

For peer review only - http://bmjopen.bmj.com/site/about/guidelines.xhtml

> *talk to people now. I mean, I'll sit out at my doorstep and, you know, a lot of people'll stop and chat to me, but I don't really like it'.* Karen, age 54, White British, non-participant

Despite scoring high on the 'Indices of Deprivation'[29], the three *Well London* intervention neighbourhoods will be described separately in acknowledgement of their diversity and to bring out the nuances of how place impacts participation and any consequent outcomes.

HARTFIELD is a large housing estate built in the 1950s, comprising low-rise blocks constructed around a series of rectangular grassed areas. Although the most homogenous of the three neighbourhoods in terms of population and environment, prior to *Well London* it was socially fragmented with a dearth of community activities.
Pre-intervention descriptions of Hartfield included:

> *'And the word 'Hartfield' put horror – it was notorious. Everybody who was difficult was dumped here'.* Margaret, 59, White British

> *It's a dumping ground. It always has been a dumping ground. You know? I begged not to be put on there. I've been there 21 years.* Liz, 48, Irish

> *I will tell you straightaway there was no life in the community before the arrival of* Well London. *No, that is the summary of the whole thing; where you are living in an area where there was no life.* Clifford, 46, African (Uganda)

Post-intervention, Hartfield respondents reported the most substantive change in experienced health benefits of all 3 neighbourhoods. Factors that facilitated this included: a) a pro-active, charismatic Well-London coordinator; b) increased safety following changes in policing methods on the estate, instigated by the coordinator; c) a high number of proactive volunteers; and d) residents as stakeholders through the estate's Residents Committee, set up by *Well London*. Benefits described included: enhanced feeling of social cohesion, new knowledge about health, involvement in estate-wide activities, improved relations with neighbours, less complaints about the neighbourhood's lack of cleanliness, safety and violence.

**Box 1 Hartfield**	**Reported benefits in HE, PA, MHWB, and Social Interaction**
**Healthy Eating**	➢ *So you know, after walk we have this exercise to stretch ourselves, and then after that we used to have fruit. Yeah, so we used to sit in the park and we used to eat fruit and that's how I learn to eat fruit basically.* Priya, 34, Indian<br>➢ *Oh my God, people are healthier now. It's changed, it's completely changed. I say that it's changed because I am involved - I know how much to my own particular health* (and*) the health of my family and how much has changed. I'm able to know more now, I know what to eat, what not to eat.* Thomas, 45, African (Ghana)

For peer review only - http://bmjopen.bmj.com/site/about/guidelines.xhtml

**Physical Activity**	➢	*Like before, you know, I used to find walking was kind of one of the painful things, yeah, I wouldn't bother to walk, I would rather take bus rather than walking, but now I feel like, now - rather than taking bus or anything, let's just walk, it's not going to take me that long.* Sandra, 43, African (Uganda)
	➢	*And we have some people who want to go night walk - like the Somalians. If the place is dark, they would like to walk. Because of, you know, night-time you can also wear your trousers - so that they can walk faster.* Joyce, 38, African (Nigeria)
**Mental Health, Wellbeing**	➢	*The fact that it's made me proud of myself and the whole project and the whole community, because it's made people come in to do the activities.* Bernard 42, African (Ivory Coast)
	➢	*It has made the community come together, that's what I've seen anyway, people have come together, which is very good.* Claudette, 37, African (Sierra Leone)
**Social interaction**	➢	*Yes we used to be on our own, nobody say hello to each other, but because of Cheryl,* Well London *came to this place* (and*) it start connecting us.* Lorraine, 39, African (Uganda)
	➢	*Yeah the police, which are responsible to this area, yes it's changed a lot because now they can say 'hello' to you. Sometimes even the kids, if they see them playing outside they will stand and speak with them, and ask them 'are you with elderly adults or are you alone?' And so forth and we are happy for that.* Margaret, 41, African (Ghana)
	➢	*I think that it does a good thing* - Well London *came to help out. We did a food basket with five foods, I did that as well so. I know the women come to do, they have sewing classes, and it's just - but it's for the coming together, the community together, that's what I think.* Clara, 42, Bangladeshi
	➢	*I've been proud to say that this is one of my proudest periods in regards to this community. Yeah, this is because our efforts that has been put in place by* Well London *and followed by* Well London *volunteers. The Hartfield estate now compared to what it was in the past, it's a name at least to be proud of.* Frank, 39, African (Nigerian)

EASTFORD has undergone extensive regeneration over the last decade, including funding to develop community projects that promote health. This had generated an ethos of community participation and differentiating the *Well London* interventions from these other activities was subsequently more difficult, especially its effects on Mental Health and Well-being. Despite this, the positive changes experienced here by participants refer specifically to the *Well London* activities.

Pre-intervention, Eastford was already defined as a place where things happened: 'Eastford *is great – there's so much to do here!'* Jermina, 26, Bangladeshi.
Post-intervention, respondents experienced some change. Benefits included a) a sense of autonomy from volunteering and involvement in managing and running activities; b) feeling

For peer review only - http://bmjopen.bmj.com/site/about/guidelines.xhtml

productive and useful; c) increased knowledge of food/cooking & improved health; and c) enhanced feeling of social cohesion.

Box 2 Eastford	Reported benefits in HE, PA, and MHWB and Social Interaction
**Healthy Eating**	➢ *Yeah, whereas say somebody comes in on Tuesday and does a little bit of cooking - it's quite quick, but with the 'Cook & Eat' it was more in depth and they explained things better and you could ask questions and all things like that, yeah, it was much better.* Clare, 38, White British<br>➢ *Earlier I used to be like, junkie foods eating; crisps and all those things. Now it's like more fruit and vegetables and salad in my diet.* Shubha, 28, Indian
**Physical Activity**	➢ *It was great, it's fantastic - I cannot express how good it is to get in there and get your hands dirty, and to see everybody else doing the same thing.* Sarah, 34, White British<br>➢ *Yes, I do a lot, because I'm doing them exercises it's helped me, it's good for my health, I feel much better, I can breathe properly. And you make friend. Yeah, it's good for me - I go out, and you meet friends.* Tricia, 72, Caribbean
**Mental Health and Well-being**	➢ *I feel I can keep my mind going and I feel like my mind has to be active because I don't want to sit down and get depressed or something. If I think bad things then I won't be doing nothing and I don't want to go like that yeah.* Maureen, 48, Irish
**Social interaction**	➢ *I feel so much more confident that we can make this move on; the thing we were given was confidence building. I think that sort of confidence building was something I didn't see – yeah, running an organisation, running that level of budgeting and planning.* Michael, 50, White British<br>➢ *You can see it, just a healthier lifestyle: people busy all the time, people - not so much arguments and you see that less and people are a lot more sociable as well.* Pat, 36, Black British

MOUNTSIDE

Mountside is a neighbourhood of contradictions, characterized by a geographically dispersed, ethnically and socio-economically diverse but transitory population and a reverse trend in terms of regeneration:

> *'It was a transient population so you'd get people move in for three months, as I say, trash the place or do whatever'*. Paula, 45, White British

> *'It became what I can only describe as a dumping ground for literally anybody. There was no perspective on who was living where and next to whom; people were just thrown into the flats regardless of background, criminal intention or anything'.* Mohan, 52, Indian

For peer review only - http://bmjopen.bmj.com/site/about/guidelines.xhtml

'When we first moved here it was gorgeous. Oh, you couldn't have wished for a more idyllic place to live. It was quiet, it was flowers, it was lovely neighbours. But of course, a lot of our neighbours then had been here since the block went first up in the '60s, so they were all getting old and consequently all started to die and then their families sold the flats to housing associations. And you go from there'. Monica, 62, White British

The loss of facilities such as a local cinema, shops and other community activities resulted in the main street consisting of fast food outlets and budget shops – high street brands that used to exist had moved away, apart from a large supermarket. There were pockets of privately owned terraced housing divided from local authority tower blocks, marking a clear socio-economic boundary. Attitudes to *Well London* were similarly divided; some viewed the interventions positively, and some not.

Post-intervention, Mountside respondents recounted little change; positive change was commented on only in relation to the Mental Health and Well-being activities. Factors that prevented change were: a) lack of effective, coordinated local leadership; b) dispersal and transience of the local population; c) lack of cohesive environmental planning; and d) strong sense of neglect and 'being forgotten' by residents.

**Box 3 Mountside**	**Reported benefits in HE, PA, and MHWB**
**Healthy Eating**	➢ *Yeah, you know children like chips, sausages, yeah. Just sometimes I'm cooking chips - every time Turkish foods; rice yeah. You know, my older one all the time she wants outside, McDonalds, chicken, chips, she's eating too much. And everywhere this food. I'm telling her 'you know, too much oily inside, you no eat' and she's not listening to me.* Hanife, 36, Turkish<br>➢ *You can see the higher fast food intake, zero exercise, high alcohol and stressful kind of lifestyles that people lead. And this is also supported by the number of fast food outlets that thrive in these areas.* Mohan, 31, Asian British
**Physical Activity**	➢ *We've got one park over the road but, again that's a dangerous place. We've had murders over there, we've had people killing the swans to eat and people sleeping rough over there. So of course, parents weren't taking their kids over to the park, and you can't blame them, I wouldn't go over there.* Marie, 46, White British
**Mental Health and Well-being**	➢ *It was fantastic, it brought up a lot of issues and a lot of practices and things that I'm already aware of, and I really, really enjoyed it'.* Saroja, 27, Asian British<br>➢ *Oh yeah, and I wish it could continue, I really, really do, because I think it's started to actually break down a few barriers. We were all really sad when it ended and I thought; this is something that could really build up. And I just wish we could have* Well London *permanently. It was a really nice thing, and because it came to this area it made us think, well we are important, it's come here. I know it came here because we were a deprived area but people are listening to us.*

For peer review only - http://bmjopen.bmj.com/site/about/guidelines.xhtml

	They're trying to do something to help us. And like I say, the worst thing is that we haven't got it (now). If you can bring it back I'd be ever so grateful and so would a lot of other people. Karen, 41, White British<br>➢ *I came away having learnt a lot more about the other women – appreciating them more, yeah, I think that's word should be put in there; appreciating other people, not just cultures but people themselves*. Molly, 45, Caribbean
**Social interaction**	➢ *It takes the form of exercise when I can be bothered. I will say I'm a bit lazy sometimes, so you'll do it and then it's like you don't want to take it on, on your own, so you do need motivation* Jan, age 36, White British

CONCLUSION

Participants described an overall positive impact from the *Well London* project activities, but the data also reveal a complex and nuanced picture of if and how outcomes were achieved with two key findings. Firstly, it shows how neighbourhood-level changes did not lead to benefits amongst those who did not participate in project activities. Secondly, the characteristics of neighbourhoods, both social and physical, were fundamental in moderating whether people participated, the nature and extent of the consequent benefits, and any reported changes in health practices. Therefore, participation is dependent on the provision of particular elements that support it; namely a socially cohesive environment in which to get to know neighbours; a safe environment that is well-maintained; access to affordable, nutritious food; a degree of autonomy that allows residents to be involved in decision-making and thereby improve confidence and self-esteem.

The role of the *Well London* co-ordinators also emerged as an important theme across the three areas. With their active involvement through co-ordinated organisation of volunteers, a commitment to the area shown by their understanding of local issues, participation was more successfully implemented. In Hartfield for example, residents frequently cited their co-ordinator; as a 'boundary crosser'[30], she was pivotal in encouraging and facilitating their involvement in activities. As a she By contrast, co-ordination of the activities in Mountside were deemed problematic by many - apart from those attending DIY Happiness groups, which were identified as positive because they acknowledged residents' sense of deprivation. Activities that did less well were those deemed to be out of touch with local needs i.e 'fun' activities were less successful than those seen to have direct relevance and benefit, such as stress management. In Eastford, residents were encouraged to lead projects and be involved in decision-making and subsequently, external *Well London* leadership and co-ordination was mentioned less, whereas the benefits of taking a leadership role were spoken of frequently.

Well-being was a central requirement for the exercise of personal agency, which in turn enabled participation in the *Well London* activities. Well-being was tied to factors such as being able to live in a socially cohesive and safe neighbourhood where neighbours respected one another and where problems were recognised and acted upon by local authorities. Once engaged there was an apparent feedback loop whereby further enhancement of well-being increased personal agency and lead to increased involvement in the activities which then lead to changes in attitudes and practices in eating, exercise and mental health. Participants' well-being, agency and participation also interacted with their sense of place, again in an iterative fashion. Following improvements to the physical environment, such as direct involvement of

Comment [JD1]: Sentence removed here

For peer review only - http://bmjopen.bmj.com/site/about/guidelines.xhtml

local police in providing safer spaces, further enhancement of well-being and agency were described. Well-being in this instance, appears to be a crucial mediator between agency, participation and improved health practices. A recent review of individual experiences of community engagement also found that active participation in community initiatives has important psychosocial benefits for participants that include enhanced feelings of personal confidence and self-esteem, as well as enhanced social relationships and social cohesion within a community[31, 32].

In this study, participation was not a simple binary variable and quantitative measures alone did not pick up the subtleties and complex variations. Our findings show that participation is a complex and dynamic process with well-being at its core, acting as a catalyst that enables participation through a related sense of personal agency. Through further enhancement of well-being and associated social cohesion, improvements in health practices were experienced, just as in its absence no benefits were recounted. However, participation was not universally desired; some reported feeling excluded from the *Well London* interventions and the subsequent changes taking place in the neighbourhood (see participant comment, pg 6), whilst others reported little interest in taking part because improving health was neither a priority nor an personal goal.
These findings also confirm that health practices are not a separate 'capsule' of behaviour[33, 34], but are embedded within particular social, cultural and physical milieus. Across the three neighbourhoods however, there was a clear gradient of change with the greatest change seen in the presence of; a) involvement and support of external organisations; b) personal and collective agency enhanced by effective leadership in project activities; c) social cohesion fostered by *Well London* activities. Where the physical and social environment remained unchanged, there was less participation and therefore fewer benefits. Also, each area reflected considerable variation in levels of maturity and self-management: each was graded in terms of what progress was possible, Mountside being at the beginning of area level change with safety and environmental pollution still an issue in contrast to Eastford, which featured a more developed and progressive attitude due to investment both financial and from the local authority. Hartfield was in the midst of significant degrees of change through both necessity and a relatively recent influx of enthusiastic residents supported by an equally enthusiastic co-ordinator.

Comment [JD2]: Sentence removed here

As others have identified, the dynamics of participation from the perspective of individual agency have been neglected[17, 18, 32]. In addressing this, our findings show participation as a dynamic and flexible process with agency at its core. Also, the community engagement approach fed back into and reinforced feelings of well-being and agency and thus encouraged and supported changes in health practices. The findings are consistent with elements of Popay's[17] proposed pathways by which community engagement leads to health outcomes, and specifically that social capital/cohesion and community empowerment are important intermediaries[17]. Additionally they point to the need for further understanding of how these interact with agency, well-being and empowerment at the individual level and in different social contexts, in order to achieve inclusive engagement with such programmes. As Popay argues[25], people's own ideas need to be incorporated fully in the design and delivery of proposed health interventions.

Acknowledgements We thank all of the study participants for giving generously of their time; the project delivery partners for their assistance; our colleagues at the University of East

For peer review only - http://bmjopen.bmj.com/site/about/guidelines.xhtml

London, University of Westminster and the London School of Hygiene and Tropical Medicine for their support and cooperation.
Author contributions: J Derges data collection, interpretation, analysis and writing; S Jain, data interpretation and analysis; A Clow, data analysis and interpretation; R Lynch, data collection, data analysis; A Renton, project lead; A Draper, data interpretation, analysis and writing.
Funding: This work was supported by the Wellcome Trust, Grant code: 083679/Z/07/Z
Competing interests: None
Ethics approval: granted by University of Westminster Ethics Committee
Data sharing statement: Anonymised interview transcripts may be obtained on request from Dr A. Draper, University of Westminster

For peer review only

REFERENCES

1. WHO: **The Jakarta Declaration on Leading Health Promotion into the 21st Century**. Fourth International Conference on Health Promotion, Jakarta, 1997.
2. Marmot M: **The Marmot Review: Fair Society, Healthy lives – Strategic Review of Health Inequalities in England post-2010**. London, 2010.
3. Whitehead M: **A typology of actions to tackle social inequalities in health**. Journal of Epidemiology and Community Health 2007;**61:**473-478.
4. Anderson A: **The Community Builder's Approach to Theory of Change: A Practical Guide to Theory Development**. New York: The Aspen Institute Roundtable on Community Change 2005.
5. Bryce J, Victora CG, Habicht JP, Black RE, et al. (MCE-IMCI Technical Advisors): **Programmatic pathways to child survival: results of a multi-country evaluation of Integrated Management of Childhood Illness**. Health Policy and Planning Suppl 2005;**1:**15-117.
6. O'Dwyer LA, Baum F, Kavanagh A, MacDougall C: **Do area-based interventions to reduce health inequalities work? A systematic review of evidence**. Critical Public Health, 2007;**17**:317-335.

7. Victora CG, Habicht J-P, Bryce J: **Evidence-based public health: moving beyond randomized trials.** American Journal of Public Health 2004;**94**:400-405.

8. Medical Research Council: **A framework for development and evaluation of RCTs for complex interventions to improve health.** London: MRC; 2000.

9. Medical Research Council: **Developing and evaluating complex interventions: new guidance.** London: MRC; 2008.

10. Hawe P , Sheill A, Riley T, Gold, L: **Methods for exploring implementation variation and local context within a cluster randomized community intervention trial**. Journal of Epidemology and Community Health 2004;**58**:788-793.

11. Ogilvie D, Cummins S Petticrew M, White M, Jones A, Wheeler K: **Assessing the evaluability of complex public health interventions: five questions for researchers, funders, and policymakers.** Milbank Q 2011,**89**:206-225.

12. Hawe P, Shiell A, Riley T: **Complex interventions: how "out of control" can a randomized controlled trial be?** BMJ 2004, **328**: 1561-1563

13. **Well London: communities working together for a healthier city**. [http://www.welllondon.org.uk]

14. Draper AK, Hewitt G, & Rifkin SB: **Chasing the Dragon: developing indicators for the assessment of community participation in health programmes**. Soc Sci Med 2010,71:1102-1109. DOI 10.1016/j.socscimed.2010.05.016

15. Milton B, Attree P, French B, Povall S, Whitehead M, Popay J: **The impact of community engagement on health and social outcomes: a systematic review**. Community Development Journal 2012;**47**(3): 316-334.

16. Preston R, Waugh H, Larkins S, Taylor J: **Community participation in rural primary health care: intervention or approach?** Australian Journal of Primary HealthCare 2010;**16**:4-16.

For peer review only - http://bmjopen.bmj.com/site/about/guidelines.xhtml

17. Popay J: **Community engagement and community development and health improvement: a background paper for NICE**. London: NICE 2006.

18. Wallerstein N: **What is the evidence on effectiveness of empowerment to improve health?** Copenhagen, Denmark, WHO Regional Office for Europe, 2006. Health Evidence Network report; http://www.euro.who.int/Document/E88086.pdf. (accessed January 2013)

19. Cleaver F: **Institutions, agency and the limitations of participatory approaches to development**. In: Hickey S, Mohan G, eds. Participation: From Tyranny to Transformation? London Zed Books, 2004:271-283.

20. Egan M, Tannahill C, Petticrew M, Thomas, S: **Psychosocial risk factors in home and community settings and their associations with population health and health inequalities: a systematic meta-review.** BMC Public Health 2008;**8**:239

21. Forde I, Raine R: **Placing the individual within a social determinants approach to health inequity**. The Lancet 2008;**122**(11):1177-1187.

22. Wall M, Hayes R, Moore D, Petticrew M, Clow A, Schmidt E, Draper A, Lock K, Lynch R, Renton A: **Evaluation of community level interventions to address social and structural determinants of health: a cluster randomised controlled trial**. BMC Public Health 2009;9:207 DOI:10.1186/1471-2458-9-207.

23. Petticrew M: **When are complex interventions 'complex'? When are simple interventions 'simple'?** Euro J Public health 2011;**21**:397-8.

24. Phillips G, Renton A, Moore DG, Bottomley C, Schmidt E, Lais S, Yu G, Wall M, Tobi P, Frostick C, Clow A, Lock K, Petticrew, Hayes R: **The Well London program – a cluster randomized trial of community engagement for improving health behaviors and mental well-being: baseline survey results.** Trials 2012 Jul 6;13:105.doi: 10.1186/1745-6215-13-195.

For peer review only - http://bmjopen.bmj.com/site/about/guidelines.xhtml

25. Popay J: **Whose theory is it anyway?** Journal of Epidemiology and Community Medicine 2006;**60**:571-572.

26. Patton MQ: **Qualitative Research and Evaluation Methods**. 3rd Edition. Sage Publications, Thousand Oaks, California 2002:236.

27. Gardner K, Chapple A: **Barriers to referral in patients with angina: a qualitative study**. BMJ 1999;319-321.

28. Spencer L, Ritchie J, O'Connor W: **Analysis: Practices, principles and processes**. In *Qualitative research practice: a guide for social science students and researchers.* Edited by J. Ritchie & J Lewis. Thousand Oaks, CA: Sage 2003:200-218.

29. **English indices of deprivation 2010 Government Report** http://www.communities.gov.uk/publications/corporate/statistics/indices2010

30. Clow, A Hamer, M: **The iceberg of social disadvantage and chronic stress: implications for public health.** Neurosci Biobehav Rev 2010;**35**:1.

31. Attree P, French B, Milton B, Povall S, Whitehead M, Popay J: **The experience of community engagement for individuals: a rapid review of the evidence**. Health and Social Care in the Community 2011;**19**:250-60.

32. Polgar S. **Health and human behaviour: areas of interest common to the social and medical social sciences**. Current Anthropology 1962;**3**:159-205

33. Fienieg B, Nierkins V, Tonkens E, Plochg T, Stronks K. **Why play an active role? A qualitative examination of lay citizens' main motives for participation in health promotion**. Health Promotion International 2012;**27**:416-26.

34. Kilpatrick S, Cheers B, Gilles M, Taylor J: **Boundary crossers, communities, and health: exploring the role of rural health professionals**. Health Place 2009;**15**:284-290

For peer review only

For peer review only - http://bmjopen.bmj.com/site/about/guidelines.xhtml

UoW WL Evaluation
Revised Interview Topic Guide
18/02/10

UoW Well London Evaluation
Topic Guide for Phase 2 Interviews

Neighbourhood

- **Can you tell me about this area?**
  - What is it like living here?
  - Do you like living here?
  - Who lives here?
  - What are your neighbours like?
  - How did you come to be living in this area?

- **How long have you been here?**
  - Where were you before?
  - How has this area changed since you've lived here?
  - What sense of belonging or attachment, if any, do you feel to the area?
  - Do you see this area as "home"?

- **Is there a lot going on in this area?**
  - What goes on?
  - Who goes?
  - Why do people go/not go?
  - What, if anything, do you participate in in the local area or community?
  - Why do, or why don't you participate in activities in the local area?
  - Do you socialise/have friends locally?

- **How safe and comfortable do you feel in this area?**
  - What are the good things about living here?
  - Do you worry about the area?
  - Have you experienced any problems with the area or the local community?

- **How healthy are people around here?**
  - Are people locally concerned about their health?
  - Is it easy to be healthy here? Do you have access to health activities?
  - Are there things about living in this area that you think are unhealthy?
  - How do you think living in this area affects your health and well being?

Health and Wellbeing

- **What are the features to being healthy?**
  - What is a healthy lifestyle?
  - Would you say you are in good health?
  - Would you say you have a healthy lifestyle?

For peer review only

- **Do you think about your health very much?**
    - What, if anything do you do to keep yourself healthy or improve your health?
    - Do you worry about your health?
    - What do you do about this worry about your health?

- **How much do you worry about things generally?**
    - Do you have a lot to worry about?
    - What do you do when you worry?
    - How optimistic or negative do you think you are as a person?
    - How does this outlook affect the way you live your life do you think?
    - What are the positive and negative things in your life?

- **Are there people you can trust and talk to if you have problems or worries?**
    - Do you have much contact with these people?
    - Do they live nearby?

<u>Well London</u>

- **Have you heard of the Well London Project or know of any Well London activities in the area?**

- **Have you participated in any Well London activities (including the community cafes)?**
    - If so, what was your experience of these?

- **Have you felt there has been any benefit to yourself or your area from the Well London Project?**

For peer review only

'Well London' and the benefits of participation: results of a qualitative study nested in a cluster randomised trial

Journal:	*BMJ Open*
Manuscript ID:	bmjopen-2013-003596.R2
Article Type:	Research
Date Submitted by the Author:	26-Jan-2014
Complete List of Authors:	Derges, Jane; University of Westminster, Life Sciences<br>Clow, Angela; University of Westminster, Psychology<br>Lynch, Rebecca; University of Westminster, Life Sciences<br>Jain, Sumeet; University of Westminster, Life Sciences<br>Phillips, Gemma; University of East London, Institute for Health and Human Development<br>Petticrew, Mark; London School of Hygiene and Tropical Medicine, Public Health Evaluation<br>Renton, Adrian; University of East London, Institute for Health and Human Development<br>Draper, Alizon; University of Westminster, Life Sciences
**Primary Subject Heading**:	Public health
Secondary Subject Heading:	Qualitative research
Keywords:	PUBLIC HEALTH, QUALITATIVE RESEARCH, SOCIAL MEDICINE

SCHOLARONE™ Manuscripts

only

'*Well London*' and the benefits of participation: results of a qualitative study nested in a cluster randomised trial

Corresponding author:

Dr Jane Derges, School of Life Sciences, University of Westminster, 115 New Cavendish Street, London W1W 6UW

dergesj@westminster.ac.uk

Phone: 020 7911 5000 ext. 3884

Fax: 020 7911 5087

Co-authors:

Prof. Angela Clow, Psychology Department, University of Westminster, London, UK

a.clow@westminster.ac.uk

Ms Rebecca Lynch, School of Life Sciences, University of Westminster, London, UK

rebecca.j.lynch@googlemail.com

Dr Sumeet Jain, School of Life Sciences, University of Westminster, London, UK

sumeet.jain@yahoo.com

Dr Gemma Phillips, Institute for Health and Human Development, University of East London, UK; Gemma.phillips@lshtm.ac.uk

Prof. Mark Petticrew, Department of Public Health, London School of Hygiene and Tropical Medicine, London, UK; Mark.Petticrew@lshtm.ac.uk

Prof. Adrian Renton, Institute for Health and Human Development, University of East London, UK; adrian2@UEL-Exchange.uel.ac.uk

Dr Alizon Draper, School of Life Sciences, University of Westminster, London, UK

a.draper@westminster.ac.uk

For peer review only

ABSTRACT

Background
Well London is a multi-component community engagement and co-production programme designed to improve the health of Londoners living in socio-economically deprived neighbourhoods. To evaluate outcomes of the *Well London* interventions, a cluster randomized trial (CRT) was conducted that included a longitudinal qualitative component, which is reported here. The aim is to explore in depth the nature of the benefits to residents and the processes by which these were achieved.

Methods
The one-year longitudinal qualitative study was nested within the CRT. Purposive sampling was used to select three intervention neighbourhoods in London and 61 individuals within these neighbourhoods. The interventions comprised activities focused on: healthy eating (HE), physical exercise (PE) and mental health and well-being (MHWB). Interviews were conducted at inception and following completion of the *Well London* interventions to establish both if and how they had participated. Transcripts of the interviews were coded and analysed using Nvivo.

Results
Positive benefits relating to the formal outcomes of the CRT were reported, but only among those who participated in project activities. The extent of benefits experienced was influenced by factors relating to the physical and social characteristics of each neighbourhood. The highest levels of change occurred in the presence of: a) social cohesion, pre-existing but also as facilitated by *Well London* activities; b) personal and collective agency; c) involvement and support of external organisations. Where the physical and social environment remained unchanged, there was less participation and fewer benefits.

Conclusion
These findings show interaction between participation, well-being and agency, social interactions and cohesion, and that this modulated any benefits described. Pathways to change were thus complex and variable, but both personal well-being and local social cohesion emerged as important mediators of change.

Key words: Public health, programme intervention evaluation, qualitative study, interviews

Article summary: strengths and limitations of the study

Strengths

- Uses participants perspectives to identify why and how people participate in community health programmes
- Focuses on agency and its relationship to 'well-being' within an urban marginalised population
- Highlights the importance of social cohesion to 'well-being' and subsequently, participation in community health programmes

Limitations

- Further exploration of non-participation is required for future study
- An ethnographic focus would contribute both methodologically and to analysis

For peer review only

BACKGROUND

Processes of change in community-based interventions

Improving the health and well-being of populations living in disadvantaged areas of the UK remains a key public health challenge[1,2]. But while there is extensive evidence documenting the consequences and causes of health inequalities, less is known about the interplay between specific causal factors or what interventions are effective in reducing them[3]. There is growing recognition of the need to understand which interventions are effective and the processes or pathways by which effects are achieved[4-7]. This is particularly important for interventions that are "complex"[8,9] and in which local contextual factors modulate both the process of implementation and generation of outcomes[10]. The *Well London* programme is a complex intervention comprising multiple components and using a community engagement model. The term 'participation' is used here to highlight participants' agency in relation to choice and whether or not they decided to take part in the interventions, including as volunteers. The interventions comprised a series of activities based around healthy eating, physical activity and mental well-being. 'Well-being' is here defined as a eudemonic state in which the individual experiences positive attachment, a sense of meaningfulness and usefulness in life. This framework was used in each area but the delivery method varied according to local needs and priorities, as outlined in current theories concerning the design and evaluation of complex interventions[11,12]. Further details are obtainable from the *Well London* Website[13].
As Draper at al. and others note there is a need for rigorous evaluations that explore the causal pathways by which community participation influences health outcomes[14-16]. Popay[17] and Wallerstein[18] have hypothesized a number of possible pathways, but these remain largely unexamined. The relationship between social context, individual agency and participation has also been neglected[19], as well as exploration of the effects of interventions to address health inequalities[20] and the nature of personal agency and its relationship to a social determinants of health framework[21]. Whilst the social and environmental context in which people live and their ability to exercise individual agency in relation to decision-making about health is recognized as important, there are few qualitative studies that examine how these are interlinked and impact community engagement programmes.

The analysis presented here focuses on a qualitative study, which was embedded within the cluster randomized trial (CRT) of the *Well London* programme (see[22] for a full description of the trial protocol). The primary aim of the qualitative study was to examine the causal pathways that generated any intervention effects from the perspectives of local residents, who were involved as strategic partners in *Well London*'s design and delivery.

METHODS

Study Objectives

The objectives of the qualitative study were:

1. To identify how and why individuals participated in *Well London* activities;
2. To explore the different project components that enabled people to improve their health practices and sense of 'well-being';
3. To identify factors in the social and physical environment that influenced attitudes to health.

Study design

A longitudinal qualitative research element was included in the CRCT in order 1) to address the complexity[23] of the intervention; 2) to try to identify specific factors which enable or

obstruct individuals in leading healthy lives; 3) to understand subjective experience and the role of 'agency' in relation to participation.

The longitudinal qualitative study component was nested within the larger *Well London* CRT (for details of overall trial design, see[24]). It comprised a series of in depth interviews conducted over a period of one year, with interviews undertaken in two stages: firstly, at the implementation stage of the interventions and secondly, post-intervention. Both participants and non-participants of *Well London* were interviewed to capture whether exposure to the interventions would lead to neighbourhood level improvements in health and health practices, or whether direct participation was required. Limited observation of three selected *Well London* intervention neighbourhoods was also undertaken, to contextualise the interview data. Interviews were inductively examined in accordance with increasing calls for better capture of participants views[25].

Selection of study neighbourhoods

Initial observation showed contextual variation between the twenty *Well London* intervention neighbourhoods in environment, demography and history as well as in intensity and range of community activities running parallel to (i.e not commissioned by) *Well London*. A critical case sampling approach was therefore used to select 3 neighbourhoods to be included in the qualitative study (chosen in consideration of the in depth nature of the study). This approach selects cases based on criteria that are seen to be particularly important for the research project: "if it happens there, it will happen everywhere" or "if it doesn't happen there it won't happen anywhere", "if that group is having problems we can be sure that every group is having problems"[26].

In selecting this approach it was necessary to identify what would make a critical case in relation to the objectives of the qualitative study. We therefore included neighbourhoods with both low and high levels of community projects. The method of programme delivery within *Well London* also differed and the three neighbourhoods chosen reflect this by including neighbourhoods with high and low levels of pre-existing community activities beyond those provided by the *Well London* programme, and differences in the manner of their delivery. The first of these neighbourhoods (Eastford[1]) had a wide range of community activities offered prior to *Well London*, and continues to offer many activities unconnected to *Well London*. The second neighbourhood (Hartfield) has a core group of volunteers instrumental in generating engagement in *Well London* activities and they live in an enclosed geographical space (a housing estate). The third neighbourhood (Mountside) had limited community activities prior to *Well London* and a population dispersed among a number of differing housing sites. The manner of *Well London* delivery in these neighbourhoods varied from highly pro-active and involved members to a less cohesive and active method of delivery.

Study population

61 individuals were recruited at the start of *Well London* delivery and comprised matched participants of the interventions, and non-participants (see Table 1). Participants were purposively selected from within the interventions across the 3 study neighbourhoods and non-participants were selected through snowball sampling; these contacts were made by the researcher during visits to the neighbourhood. Selection for the qualitative study was based on providing theoretical insights rather than broader generalisations, as noted by Gardner and Chapple[27]. 'Participant' is here defined as residents who both received the *Well London* activities, and volunteered in their delivery.

[1] Pseudonyms are used throughout for places and people

For peer review only - http://bmjopen.bmj.com/site/about/guidelines.xhtml

For the post-intervention interview, a total of 45 agreed to a second interview. Reasons given by the 16 who did not attend this second interview were as follows: moved out of the neighbourhood (2), refusal of a follow-up interview (3), no response elicited (9), and illness (2). New recruits were not sought as changes over the intervening period would not have been captured.
Ethnicity, age and length of time in the neighbourhood among the study population were mixed across all three neighbourhoods; each neighbourhood showed variation according to all these categories, most noticeably, ethnicity. It is beyond the scope of this paper to examine the effects of this in detail, other than to recognize this as a difference requiring further investigation.

Table 1. Participant and non-participant profiles

1st Round interviews	Age range	Ethnicity	Gender	*Well London* participation	2nd Round interviews
Hartfield	16-25: 3<br>26-35: 8<br>36-45: 3<br>46-55: 5<br>56-65: 2<br>66-89: 1	African 13, Indian 3, Bangladeshi 1, White British 3, European (Lithuania) 1	Female: 16<br>Male: 5	Participants: 13<br>Non-participant: 8<br>Total: 21	Stage 2.<br>P=11<br>NP=8<br>Total: 20
Eastford	16-25: 4<br>26-35: 4<br>36-45: 2<br>46-55: 4<br>56-65: 3<br>66-75: 3	Bangladeshi 5, Indian 1, Pakistani 1, Caribbean 2, Black British 1, African 3, Chinese 2, White British 3, Irish 2	Female: 18<br>Male: 2	Participants: 11<br>Non-participants: 9<br>Total: 20	Stage 2.<br>P=7<br>NP=8<br>Total: 15
Mountside	16-25: 5<br>26-35: 3<br>36-45: 4<br>46-55: 3<br>56-65: 3<br>66-75: 2	White British 5, British Asian 5, Caribbean 4, European (Turkish) 3, African 2, Chinese 1	Female: 11<br>Male: 9	Participants: 10<br>Non-participant: 10<br>Total: 20	Stage 2.<br>P=6<br>NP=4<br>Total: 10

Data collection and analysis
Pre- and post intervention interviews used the same topic guide and focused on participants experiences of the *Well London* interventions and any reported changes to eating, exercise and mental health practices (see supplementary file). Also both participants and non-participants were asked for their views about the neighbourhood environment. Interviews were recorded and transcribed before being entered into Nvivo. Each transcript was checked for quality, coded and analysed using a framework based on Spencer, Ritchie and O'Conner's 'analytic hierarchy'[28]). This allowed systematic analysis of the large dataset but was flexible enough to allow refinements to the coding. Codes from interviews were identified and grouped under categories generated from the interview topics. Data were analysed not only deductively from the primary outcome measures (changes to healthy eating, physical activity and mental well-being), but also inductively from emerging themes identified from the interviews.
Observational data of the neighbourhoods was recorded both in photographs and written notes and included the local geography, amenities and range of community facilities and activities that were available. A separate researcher was employed at each stage of the study;

one researcher for stage 1 interviews, and the second researcher for stage 2. They each also conducted the observations simultaneous to the interviews.

Interview quality assurance and ethics

Quality assurance procedures were undertaken to minimise researcher bias when coding the interviews by randomly selecting three interviews, which were then recoded by two independent researchers blind to the initial coding. The three interviews were compared to identify new codes and establish a degree of consensus in applying a particular code to similar text. The University of Westminster Research Ethics Committee approved the study. Following initial telephone/email contact, written consent was obtained from every participant/non-participant. Each individual also received a short pamphlet describing the *Well London* project and an explanation of the qualitative component. Verbal explanation was also provided at the start of the interview process.

The results of the qualitative study are presented here by area. The reason for this is that the context and environment in which participants and non-participants were living and into which the *Well London* interventions and activities were introduced, has been shown to be a key factor in showing why individuals participated. Subsequently, data are presented by area, not theme.

RESULTS

Findings from the qualitative data show participants describing positive changes, both to their individual health and experiences of their neighbourhood as a result of participation in the *Well London* activities. However, equally significant was the degree of variation in how these changes were perceived between each neighbourhood, which was modulated by: mode of delivery, characteristics of individuals, neighbourhood history and attitudes to social interaction. As a consequence, each area is described separately in the results and the basis for using quotes is to represent what was said in relation to the themes.

Overall, participants identified the importance of social interaction as a crucial component of participation in the *Well London* activities. For example: a social gathering that included eating healthy food; gardening and opportunities to chat over a cup of tea; women feeling safer when sharing an evening walk together. Participation in practical health-related activities were only beneficial within a social context. By comparison, non-participants, despite their individual attempts at improving their own health, experienced no benefits either from efforts to change eating or physical activity levels, or from being around others in the community who were participating in *Well London* activities:

> *'So I was left a very lonely bunny for quite a while. I don't like going out walking all the time on my own, I don't like going swimming on my own. I love to do it, but I don't like doing it on my own. If there was a group going, I would go'* Mary, age 48, Irish, non-participant

Furthermore, a small number of non-participants felt excluded from the *Well London* interventions, suggesting there may be some non-beneficial effects. For example, in response to a question about positive changes on the estate as a result of *Well London*, one resident commented:

> *'Well, that "getting better" is a matter of opinion, because I look on it now as a ghetto. It was an unruly estate before. It has quietened down, but now it's a ghetto. I don't go out anymore, I don't do anything anymore. No. No. And I hardly even*

For peer review only

talk to people now. I mean, I'll sit out at my doorstep and, you know, a lot of people'll stop and chat to me, but I don't really like it'. Karen, age 54, White British, non-participant

Despite scoring high on the 'Indices of Deprivation'[29], the three *Well London* intervention neighbourhoods will be described separately in acknowledgement of their diversity and to bring out the nuances of how place impacts participation and any consequent outcomes.

HARTFIELD is a large housing estate built in the 1950s, comprising low-rise blocks constructed around a series of rectangular grassed areas. Although the most homogenous of the three neighbourhoods in terms of population and environment, prior to *Well London* it was socially fragmented with a dearth of community activities.
Pre-intervention descriptions of Hartfield included:

'And the word 'Hartfield' put horror – it was notorious. Everybody who was difficult was dumped here'. Margaret, 59, White British

It's a dumping ground. It always has been a dumping ground. You know? I begged not to be put on there. I've been there 21 years. Liz, 48, Irish

I will tell you straightaway there was no life in the community before the arrival of Well London*. No, that is the summary of the whole thing; where you are living in an area where there was no life.* Clifford, 46, African (Uganda)

Post-intervention, Hartfield respondents reported the most substantive change in experienced health benefits of all 3 neighbourhoods. Factors that facilitated this included: a) a pro-active, charismatic Well-London coordinator; b) increased safety following changes in policing methods on the estate, instigated by the coordinator; c) a high number of proactive volunteers; and d) residents as stakeholders through the estate's Residents Committee, set up by *Well London*. Benefits described included: enhanced feeling of social cohesion, new knowledge about health, involvement in estate-wide activities, improved relations with neighbours, less complaints about the neighbourhood's lack of cleanliness, safety and violence (See box 1; social interaction).

**Box 1 Hartfield**	**Reported benefits in HE, PA, MHWB, and Social Interaction**
**Healthy Eating**	➢ *So you know, after walk we have this exercise to stretch ourselves, and then after that we used to have fruit. Yeah, so we used to sit in the park and we used to eat fruit and that's how I learn to eat fruit basically.* Priya, 34, Indian<br>➢ *Oh my God, people are healthier now. It's changed, it's completely changed. I say that it's changed because I am involved - I know how much to my own particular health* (and*) the health of my family and how much has changed. I'm able to know more now, I know what to eat, what not to eat.* Thomas, 45, African (Ghana)

**Physical Activity**	➢ *Like before, you know, I used to find walking was kind of one of the painful things, yeah, I wouldn't bother to walk, I would rather take bus rather than walking, but now I feel like, now - rather than taking bus or anything, let's just walk, it's not going to take me that long.* Sandra, 43, African (Uganda)<br>➢ *And we have some people who want to go night walk - like the Somalians. If the place is dark, they would like to walk. Because of, you know, night-time you can also wear your trousers - so that they can walk faster.* Joyce, 38, African (Nigeria)
**Mental Health, Wellbeing**	➢ *The fact that it's made me proud of myself and the whole project and the whole community, because it's made people come in to do the activities.* Bernard 42, African (Ivory Coast)<br>➢ *It has made the community come together, that's what I've seen anyway, people have come together, which is very good.* Claudette, 37, African (Sierra Leone)
**Social interaction**	➢ *Yes we used to be on our own, nobody say hello to each other, but because of Cheryl* (*Well London* co-ordinator)*,* Well London *came to this place* (and) *it start connecting us.* Lorraine, 39, African (Uganda)<br>➢ *Yeah the police, which are responsible to this area, yes it's changed a lot because now they can say 'hello' to you. Sometimes even the kids, if they see them playing outside they will stand and speak with them, and ask them 'are you with elderly adults or are you alone?' And so forth and we are happy for that.* Margaret, 41, African (Ghana)<br>➢ *I think that it does a good thing* - Well London *came to help out. We did a food basket with five foods, I did that as well so. I know the women come to do, they have sewing classes, and it's just - but it's for the coming together, the community together, that's what I think.* Clara, 42, Bangladeshi<br>➢ *I've been proud to say that this is one of my proudest periods in regards to this community. Yeah, this is because our efforts that has been put in place by* Well London *and followed by* Well London *volunteers. Last year I was just a volunteer to* Well London *but this year I am the chairman of Hartfield Estate - the estate now compared to what it was in the past, it's a name at least to be proud of.* Frank, 39, African (Nigerian)<br>➢ *Before we started living here I heard (that) the estate wasn't really nice, it wasn't really good. Yeah, in terms of gangs and all those things. But I was, well initially I was a bit scared…no this place has changed now. It's not like the way it used to be before* (Well London)*, there's a lot of cameras around, and then there's this local police office behind, just around there, which is really good, so.*

EASTFORD has undergone extensive regeneration over the last decade, including funding to develop community projects that promote health. This had generated an ethos of community participation and differentiating the *Well London* interventions from these other activities was subsequently more difficult, especially its effects on Mental Health and Well-being. Despite this, the positive changes experienced here by participants refer specifically to the *Well London* activities.

Pre-intervention, Eastford was already defined as a place where things happened: 'Eastford *is great – there's so much to do here!'* Jermina, 26, Bangladeshi.
Post-intervention, respondents experienced some change. Benefits included a) a sense of autonomy from volunteering and involvement in managing and running activities; b) feeling productive and useful; c) increased knowledge of food/cooking & improved health; and c) enhanced feeling of social cohesion.

Box 2 Eastford	Reported benefits in HE, PA, and MHWB and Social Interaction
**Healthy Eating**	➢ *Yeah, whereas say somebody comes in on Tuesday and does a little bit of cooking - it's quite quick, but with the 'Cook & Eat' it was more in depth and they explained things better and you could ask questions and all things like that, yeah, it was much better.* Clare, 38, White British<br>➢ *Earlier I used to be like, junkie foods eating; crisps and all those things. Now it's like more fruit and vegetables and salad in my diet.* Shubha, 28, Indian
**Physical Activity**	➢ *It was great, it's fantastic - I cannot express how good it is to get in there and get your hands dirty, and to see everybody else doing the same thing.* Sarah, 34, White British<br>➢ *Yes, I do a lot, because I'm doing them exercises it's helped me, it's good for my health, I feel much better, I can breathe properly. And you make friend. Yeah, it's good for me - I go out, and you meet friends.* Tricia, 72, Caribbean
**Mental Health and Well-being**	➢ *I feel I can keep my mind going and I feel like my mind has to be active because I don't want to sit down and get depressed or something. If I think bad things then I won't be doing nothing and I don't want to go like that yeah.* Maureen, 48, Irish
**Social interaction**	➢ *I feel so much more confident that we can make this move on; the thing we were given was confidence building. I think that sort of confidence building was something I didn't see – yeah, running an organisation, running that level of budgeting and planning.* Michael, 50, White British<br>➢ *You can see it, just a healthier lifestyle: people busy all the time, people - not so much arguments and you see that less and people are a lot more sociable as well.* Pat, 36, Black British

MOUNTSIDE

For peer review only - http://bmjopen.bmj.com/site/about/guidelines.xhtml

Mountside is a neighbourhood of contradictions, characterized by a geographically dispersed, ethnically and socio-economically diverse but transitory population and a reverse trend in terms of regeneration:

> *'It was a transient population so you'd get people move in for three months, as I say, trash the place or do whatever'*. Paula, 45, White British

> *'It became what I can only describe as a dumping ground for literally anybody. There was no perspective on who was living where and next to whom; people were just thrown into the flats regardless of background, criminal intention or anything'*. Mohan, 52, Indian

> *'When we first moved here it was gorgeous. Oh, you couldn't have wished for a more idyllic place to live. It was quiet, it was flowers, it was lovely neighbours. But of course, a lot of our neighbours then had been here since the block went first up in the '60s, so they were all getting old and consequently all started to die and then their families sold the flats to housing associations. And you go from there'*. Monica, 62, White British

The loss of facilities such as a local cinema, shops and other community activities resulted in the main street consisting of fast food outlets and budget shops – high street brands that used to exist had moved away, apart from a large supermarket. There were pockets of privately owned terraced housing divided from local authority tower blocks, marking a clear socio-economic boundary. Attitudes to *Well London* were similarly divided; some viewed the interventions positively, and some not.

Post-intervention, Mountside respondents recounted little change; positive change was commented on only in relation to the Mental Health and Well-being activities. Factors that prevented change were: a) lack of effective, coordinated local leadership; b) dispersal and transience of the local population; c) lack of cohesive environmental planning; and d) strong sense of neglect and 'being forgotten' by residents.

**Box 3 Mountside**	**Reported benefits in HE, PA, and MHWB**
**Healthy Eating**	➢ *Yeah, you know children like chips, sausages, yeah. Just sometimes I'm cooking chips - every time Turkish foods; rice yeah. You know, my older one all the time she wants outside, McDonalds, chicken, chips, she's eating too much. And everywhere this food. I'm telling her 'you know, too much oily inside, you no eat' and she's not listening to me.* Hanife, 36, Turkish<br>➢ *You can see the higher fast food intake, zero exercise, high alcohol and stressful kind of lifestyles that people lead. And this is also supported by the number of fast food outlets that thrive in these areas.* Mohan, 31, Asian British
**Physical Activity**	➢ *We've got one park over the road but, again that's a dangerous place. We've had murders over there, we've had people killing the swans to eat and people sleeping rough over there. So of course, parents weren't taking their kids over to the park, and you can't blame them, I wouldn't go over there.* Marie, 46, White British

Mental Health and Well-being	➢	*It was fantastic, it brought up a lot of issues and a lot of practices and things that I'm already aware of, and I really, really enjoyed it'.* Saroja, 27, Asian British
	➢	*Oh yeah, and I wish it could continue, I really, really do, because I think it's started to actually break down a few barriers. We were all really sad when it ended and I thought; this is something that could really build up. And I just wish we could have* Well London *permanently. It was a really nice thing, and because it came to this area it made us think, well we are important, it's come here. I know it came here because we were a deprived area but people are listening to us. They're trying to do something to help us. And like I say, the worst thing is that we haven't got it* (now)*. If you can bring it back I'd be ever so grateful and so would a lot of other people.* Karen, 41, White British
	➢	*I came away having learnt a lot more about the other women – appreciating them more, yeah, I think that's word should be put in there; appreciating other people, not just cultures but people themselves*. Molly, 45, Caribbean
Social interaction	➢	*It takes the form of exercise when I can be bothered. I will say I'm a bit lazy sometimes, so you'll do it and then it's like you don't want to take it on, on your own, so you do need motivation* Jan, age 36, White British

CONCLUSION

Participants described an overall positive impact from the *Well London* project activities, but the data also reveal a complex and nuanced picture of if and how outcomes were achieved with two key findings. Firstly, it shows how neighbourhood-level changes did not lead to benefits amongst those who did not participate in project activities. Secondly, the characteristics of neighbourhoods, both social and physical, were fundamental in moderating whether people participated, the nature and extent of the consequent benefits, and any reported changes in health practices. Therefore, participation is dependent on the provision of particular elements that support it; namely a socially cohesive environment in which to get to know neighbours; a safe environment that is well-maintained; access to affordable, nutritious food; a degree of autonomy that allows residents to be involved in decision-making and thereby improve confidence and self-esteem. These findings are substantiated through the statements of participants and shown throughout, in comments concerning the importance of friendships made, improvements to eating habits, and increased feelings of safety, post *Well London*.

The role of the *Well London* co-ordinators also emerged as an important theme across the three areas. With their active involvement through co-ordinated organisation of volunteers, a commitment to the area shown by their understanding of local issues, participation was more successfully implemented. In Hartfield for example, residents frequently cited their co-ordinator; as a 'boundary crosser'[30], she was pivotal in encouraging and facilitating their involvement in activities. As a she By contrast, co-ordination of the activities in Mountside were deemed problematic by many - apart from those attending DIY Happiness groups, which were identified as positive because they acknowledged residents' sense of deprivation. Activities that did less well were those deemed to be out of touch with local needs i.e 'fun'

For peer review only - http://bmjopen.bmj.com/site/about/guidelines.xhtml

activities were less successful than those seen to have direct relevance and benefit, such as stress management. In Eastford, residents were encouraged to lead projects and be involved in decision-making and subsequently, external *Well London* leadership and co-ordination was mentioned less, whereas the benefits of taking a leadership role were spoken of frequently.

Well-being was a central requirement for the exercise of personal agency, which in turn enabled participation in the *Well London* activities. Well-being was tied to factors such as being able to live in a socially cohesive and safe neighbourhood where neighbours respected one another and where problems were recognised and acted upon by local authorities. Once engaged there was an apparent feedback loop whereby further enhancement of well-being increased personal agency and lead to increased involvement in the activities which then lead to changes in attitudes and practices in eating, exercise and mental health. Participants' well-being, agency and participation also interacted with their sense of place, again in an iterative fashion. Following improvements to the physical environment, such as direct involvement of local police in providing safer spaces, further enhancement of well-being and agency were described. Well-being in this instance, appears to be a crucial mediator between agency, participation and improved health practices. A recent review of individual experiences of community engagement also found that active participation in community initiatives has important psychosocial benefits for participants that include enhanced feelings of personal confidence and self-esteem, as well as enhanced social relationships and social cohesion within a community[31, 32].

In this study, participation was not a simple binary variable and quantitative measures alone did not pick up the subtleties and complex variations. Our findings show that participation is a complex and dynamic process with well-being at its core, acting as a catalyst that enables participation through a related sense of personal agency. Through further enhancement of well-being and associated social cohesion, improvements in health practices were experienced, just as in its absence no benefits were recounted. However, participation was not universally desired; some reported feeling excluded from the *Well London* interventions and the subsequent changes taking place in the neighbourhood (see participant comment, pg 6), whilst others reported little interest in taking part because improving health was neither a priority nor an personal goal.
These findings also confirm that health practices are not a separate 'capsule' of behaviour[33, 34], but are embedded within particular social, cultural and physical milieus. Across the three neighbourhoods however, there was a clear gradient of change with the greatest change seen in the presence of; a) involvement and support of external organisations; b) personal and collective agency enhanced by effective leadership in project activities; c) social cohesion fostered by *Well London* activities. Where the physical and social environment remained unchanged, there was less participation and therefore fewer benefits. Also, each area reflected considerable variation in levels of maturity and self-management: each was graded in terms of what progress was possible, Mountside being at the beginning of area level change with safety and environmental pollution still an issue in contrast to Eastford, which featured a more developed and progressive attitude due to investment both financial and from the local authority. Hartfield was in the midst of significant degrees of change through both necessity and a relatively recent influx of enthusiastic residents supported by an equally enthusiastic co-ordinator.

As others have identified, the dynamics of participation from the perspective of individual agency have been neglected[17, 18, 32]. In addressing this, our findings show participation as a dynamic and flexible process with agency at its core. Also, the community engagement approach fed back into and reinforced feelings of well-being and agency and thus encouraged

and supported changes in health practices. The findings are consistent with elements of Popay's[17] proposed pathways by which community engagement leads to health outcomes, and specifically that social capital/cohesion and community empowerment are important intermediaries[17]. Additionally they point to the need for further understanding of how these interact with agency, well-being and empowerment at the individual level and in different social contexts, in order to achieve inclusive engagement with such programmes. As Popay argues[25], people's own ideas need to be incorporated fully in the design and delivery of proposed health interventions.

For peer review only

For peer review only - http://bmjopen.bmj.com/site/about/guidelines.xhtml

Acknowledgements We thank all of the study participants for giving generously of their time; the project delivery partners for their assistance; our colleagues at the University of East London, University of Westminster and the London School of Hygiene and Tropical Medicine for their support and cooperation.

Funding: This work was supported by the Wellcome Trust, Grant code: 083679/Z/07/Z

Author contributions: J Derges data collection, interpretation, analysis and writing; S Jain, data interpretation and analysis; A Clow, data analysis and interpretation; R Lynch, data collection, data analysis; A Renton, project lead; A Draper, data interpretation, analysis and writing.

Competing interests: None

Ethics approval: Granted by University of Westminster Ethics Committee

Data sharing statement: Anonymised interview transcripts may be obtained on request from Dr A. Draper, University of Westminster

For peer review only

REFERENCES

1. WHO: **The Jakarta Declaration on Leading Health Promotion into the 21st Century**. Fourth International Conference on Health Promotion, Jakarta, 1997.
2. Marmot M: **The Marmot Review: Fair Society, Healthy lives – Strategic Review of Health Inequalities in England post-2010**. London, 2010.
3. Whitehead M: **A typology of actions to tackle social inequalities in health**. Journal of Epidemiology and Community Health 2007;**61:**473-478.
4. Anderson A: **The Community Builder's Approach to Theory of Change: A Practical Guide to Theory Development**. New York: The Aspen Institute Roundtable on Community Change 2005.
5. Bryce J, Victora CG, Habicht JP et al. (MCE-IMCI Technical Advisors): **Programmatic pathways to child survival: results of a multi-country evaluation of Integrated Management of Childhood Illness**. Health Policy and Planning Suppl 2005;**1:**15-117.
6. O'Dwyer LA, Baum F, Kavanagh A, et al: **Do area-based interventions to reduce health inequalities work? A systematic review of evidence**. Critical Public Health, 2007;**17**:317-335.
7. Victora CG, Habicht J-P, Bryce J: **Evidence-based public health: moving beyond randomized trials.** American Journal of Public Health 2004;**94**:400-405.
8. Medical Research Council: **A framework for development and evaluation of RCTs for complex interventions to improve health.** London: MRC; 2000.
9. Medical Research Council: **Developing and evaluating complex interventions: new guidance.** London: MRC; 2008.
10. Hawe P , Sheill A, Riley T, et al: **Methods for exploring implementation variation and local context within a cluster randomized community intervention trial**. Journal of Epidemology and Community Health 2004;**58**:788-793.

For peer review only

11. Ogilvie D, Cummins S Petticrew M, et al: **Assessing the evaluability of complex public health interventions: five questions for researchers, funders, and policymakers.** Milbank Q 2011,**89**:206-225.

12. Hawe P, Shiell A, Riley T: **Complex interventions: how "out of control" can a randomized controlled trial be?** BMJ 2004, **328**: 1561-1563

13. **Well London: communities working together for a healthier city**. [http://www.welllondon.org.uk]

14. Draper AK, Hewitt G, & Rifkin SB: **Chasing the Dragon: developing indicators for the assessment of community participation in health programmes**. Soc Sci Med 2010,71:1102-1109. DOI 10.1016/j.socscimed.2010.05.016

15. Milton B, Attree P, French B, et al: **The impact of community engagement on health and social outcomes: a systematic review**. Community Development Journal 2012;**47**(3): 316-334.

16. Preston R, Waugh H, Larkins S, et al: **Community participation in rural primary health care: intervention or approach?** Australian Journal of Primary HealthCare 2010;**16**:4-16.

17. Popay J: **Community engagement and community development and health improvement: a background paper for NICE**. London: NICE 2006.

18. Wallerstein N: **What is the evidence on effectiveness of empowerment to improve health?** Copenhagen, Denmark, WHO Regional Office for Europe, 2006. Health Evidence Network report; http://www.euro.who.int/Document/E88086.pdf. (accessed January 2013)

19. Cleaver F: **Institutions, agency and the limitations of participatory approaches to development**. In: Hickey S, Mohan G, eds. Participation: From Tyranny to Transformation? London Zed Books, 2004:271-283.

For peer review only

20. Egan M, Tannahill C, Petticrew M, et al: **Psychosocial risk factors in home and community settings and their associations with population health and health inequalities: a systematic meta-review.** BMC Public Health 2008;**8**:239

21. Forde I, Raine R: **Placing the individual within a social determinants approach to health inequity**. The Lancet 2008;**122**(11):1177-1187.

22. Wall M, Hayes R, Moore D, et al: **Evaluation of community level interventions to address social and structural determinants of health: a cluster randomised controlled trial**. BMC Public Health 2009;9:207 DOI:10.1186/1471-2458-9-207.

23. Petticrew M: **When are complex interventions 'complex'? When are simple interventions 'simple'?** Euro J Public health 2011;**21**:397-8.

24. Phillips G, Renton A, Moore DG, et al: **The Well London program – a cluster randomized trial of community engagement for improving health behaviors and mental well-being: baseline survey results.** Trials 2012 Jul 6;13:105.doi: 10.1186/1745-6215-13-195.

25. Popay J: **Whose theory is it anyway?** Journal of Epidemiology and Community Medicine 2006;**60**:571-572.

26. Patton MQ: **Qualitative Research and Evaluation Methods**. 3rd Edition. Sage Publications, Thousand Oaks, California 2002:236.

27. Gardner K, Chapple A: **Barriers to referral in patients with angina: a qualitative study**. BMJ 1999;319-321.

28. Spencer L, Ritchie J, O'Connor W: **Analysis: Practices, principles and processes**. In *Qualitative research practice: a guide for social science students and researchers.* Edited by J. Ritchie & J Lewis. Thousand Oaks, CA: Sage 2003:200-218.

29. **English indices of deprivation 2010 Government Report** http://www.communities.gov.uk/publications/corporate/statistics/indices2010

For peer review only

30. Clow, A Hamer, M: **The iceberg of social disadvantage and chronic stress: implications for public health.** Neurosci Biobehav Rev 2010;**35**:1.

31. Attree P, French B, Milton B, et al: **The experience of community engagement for individuals: a rapid review of the evidence**. Health and Social Care in the Community 2011;**19**:250-60.

32. Polgar S. **Health and human behaviour: areas of interest common to the social and medical social sciences**. Current Anthropology 1962;**3**:159-205

33. Fienieg B, Nierkins V, Tonkens E, et al. **Why play an active role? A qualitative examination of lay citizens' main motives for participation in health promotion**. Health Promotion International 2012;**27**:416-26.

34. Kilpatrick S, Cheers B, Gilles M, et al: **Boundary crossers, communities, and health: exploring the role of rural health professionals**. Health Place 2009;**15**:284-290

For peer review only

‘*Well London*’ and the benefits of participation: results of a qualitative study nested in a cluster randomised trial

Corresponding author:

Dr Jane Derges, School of Life Sciences, University of Westminster, 115 New Cavendish Street, London W1W 6UW

dergesj@westminster.ac.uk

Phone: 020 7911 5000 ext. 3884

Fax: 020 7911 5087

Co-authors:

Prof. Angela Clow, Psychology Department, University of Westminster, London, UK

a.clow@westminster.ac.uk

Ms Rebecca Lynch, School of Life Sciences, University of Westminster, London, UK

rebecca.j.lynch@googlemail.com

Dr Sumeet Jain, School of Life Sciences, University of Westminster, London, UK

sumeet.jain@yahoo.com

Dr Gemma Phillips, Institute for Health and Human Development, University of East London, UK; Gemma.phillips@lshtm.ac.uk

Prof. Mark Petticrew, Department of Public Health, London School of Hygiene and Tropical Medicine, London, UK; Mark.Petticrew@lshtm.ac.uk

Prof. Adrian Renton, Institute for Health and Human Development, University of East London, UK; adrian2@UEL-Exchange.uel.ac.uk

Dr Alizon Draper, School of Life Sciences, University of Westminster, London, UK

a.draper@westminster.ac.uk

For peer review only - http://bmjopen.bmj.com/site/about/guidelines.xhtml

ABSTRACT

Background
Well London is a multi-component community engagement and co-production programme designed to improve the health of Londoners living in socio-economically deprived neighbourhoods. To evaluate outcomes of the *Well London* interventions, a cluster randomized trial (CRT) was conducted that included a longitudinal qualitative component, which is reported here. The aim is to explore in depth the nature of the benefits to residents and the processes by which these were achieved.

Methods
The one-year longitudinal qualitative study was nested within the CRT. Purposive sampling was used to select three intervention neighbourhoods in London and 61 individuals within these neighbourhoods. The interventions comprised activities focused on: healthy eating (HE), physical exercise (PE) and mental health and well-being (MHWB). Interviews were conducted at inception and following completion of the *Well London* interventions to establish both if and how they had participated. Transcripts of the interviews were coded and analysed using Nvivo.

Results
Positive benefits relating to the formal outcomes of the CRT were reported, but only among those who participated in project activities. The extent of benefits experienced was influenced by factors relating to the physical and social characteristics of each neighbourhood. The highest levels of change occurred in the presence of: a) social cohesion, pre-existing but also as facilitated by *Well London* activities; b) personal and collective agency; c) involvement and support of external organisations. Where the physical and social environment remained unchanged, there was less participation and fewer benefits.

Conclusion
These findings show interaction between participation, well-being and agency, social interactions and cohesion, and that this modulated any benefits described. Pathways to change were thus complex and variable, but both personal well-being and local social cohesion emerged as important mediators of change.

Key words: Public health, programme intervention evaluation, qualitative study, interviews

Article summary: strengths and limitations of the study

Strengths
- Uses participants perspectives to identify why and how people participate in community health programmes
- Focuses on agency and its relationship to 'well-being' within an urban marginalised population
- Highlights the importance of social cohesion to 'well-being' and subsequently, participation in community health programmes

Limitations
- Further exploration of non-participation is required for future study
- An ethnographic focus would contribute both methodologically and to analysis

For peer review only - http://bmjopen.bmj.com/site/about/guidelines.xhtml

BACKGROUND

Processes of change in community-based interventions

Improving the health and well-being of populations living in disadvantaged areas of the UK remains a key public health challenge[1,2]. But while there is extensive evidence documenting the consequences and causes of health inequalities, less is known about the interplay between specific causal factors or what interventions are effective in reducing them[3]. There is growing recognition of the need to understand which interventions are effective and the processes or pathways by which effects are achieved[4-7]. This is particularly important for interventions that are "complex"[8,9] and in which local contextual factors modulate both the process of implementation and generation of outcomes[10]. The *Well London* programme is a complex intervention comprising multiple components and using a community engagement model. The term 'participation' is used here to highlight participants' agency in relation to choice and whether or not they decided to take part in the interventions, including as volunteers. The interventions comprised a series of activities based around healthy eating, physical activity and mental well-being. 'Well-being' is here defined as a eudemonic state in which the individual experiences positive attachment, a sense of meaningfulness and usefulness in life. This framework was used in each area but the delivery method varied according to local needs and priorities, as outlined in current theories concerning the design and evaluation of complex interventions[11,12]. Further details are obtainable from the *Well London* Website[13].
As Draper at al. and others note there is a need for rigorous evaluations that explore the causal pathways by which community participation influences health outcomes[14-16]. Popay[17] and Wallerstein[18] have hypothesized a number of possible pathways, but these remain largely unexamined. The relationship between social context, individual agency and participation has also been neglected[19], as well as exploration of the effects of interventions to address health inequalities[20] and the nature of personal agency and its relationship to a social determinants of health framework[21]. Whilst the social and environmental context in which people live and their ability to exercise individual agency in relation to decision-making about health is recognized as important, there are few qualitative studies that examine how these are interlinked and impact community engagement programmes.

The analysis presented here focuses on a qualitative study, which was embedded within the cluster randomized trial (CRT) of the *Well London* programme (see[22] for a full description of the trial protocol). The primary aim of the qualitative study was to examine the causal pathways that generated any intervention effects from the perspectives of local residents, who were involved as strategic partners in *Well London*'s design and delivery.

METHODS

Study Objectives

The objectives of the qualitative study were:

1. To identify how and why individuals participated in *Well London* activities;
2. To explore the different project components that enabled people to improve their health practices and sense of 'well-being';
3. To identify factors in the social and physical environment that influenced attitudes to health.

Study design

A longitudinal qualitative research element was included in the CRCT in order 1) to address the complexity[23] of the intervention; 2) to try to identify specific factors which enable or

For peer review only - http://bmjopen.bmj.com/site/about/guidelines.xhtml

obstruct individuals in leading healthy lives; 3) to understand subjective experience and the role of 'agency' in relation to participation.

The longitudinal qualitative study component was nested within the larger *Well London* CRT (for details of overall trial design, see[24]). It comprised a series of in depth interviews conducted over a period of one year, with interviews undertaken in two stages: firstly, at the implementation stage of the interventions and secondly, post-intervention. Both participants and non-participants of *Well London* were interviewed to capture whether exposure to the interventions would lead to neighbourhood level improvements in health and health practices, or whether direct participation was required. Limited observation of three selected *Well London* intervention neighbourhoods was also undertaken, to contextualise the interview data. Interviews were inductively examined in accordance with increasing calls for better capture of participants views[25].

Selection of study neighbourhoods

Initial observation showed contextual variation between the twenty *Well London* intervention neighbourhoods in environment, demography and history as well as in intensity and range of community activities running parallel to (i.e not commissioned by) *Well London*. A critical case sampling approach was therefore used to select 3 neighbourhoods to be included in the qualitative study (chosen in consideration of the in depth nature of the study). This approach selects cases based on criteria that are seen to be particularly important for the research project: "if it happens there, it will happen everywhere" or "if it doesn't happen there it won't happen anywhere", "if that group is having problems we can be sure that every group is having problems"[26].

In selecting this approach it was necessary to identify what would make a critical case in relation to the objectives of the qualitative study. We therefore included neighbourhoods with both low and high levels of community projects. The method of programme delivery within *Well London* also differed and the three neighbourhoods chosen reflect this by including neighbourhoods with high and low levels of pre-existing community activities beyond those provided by the *Well London* programme, and differences in the manner of their delivery.

The first of these neighbourhoods (Eastford[1]) had a wide range of community activities offered prior to *Well London*, and continues to offer many activities unconnected to *Well London*. The second neighbourhood (Hartfield) has a core group of volunteers instrumental in generating engagement in *Well London* activities and they live in an enclosed geographical space (a housing estate). The third neighbourhood (Mountside) had limited community activities prior to *Well London* and a population dispersed among a number of differing housing sites. The manner of *Well London* delivery in these neighbourhoods varied from highly pro-active and involved members to a less cohesive and active method of delivery.

Study population

61 individuals were recruited at the start of *Well London* delivery and comprised matched participants of the interventions, and non-participants (see Table 1). Participants were purposively selected from within the interventions across the 3 study neighbourhoods and non-participants were selected through snowball sampling; these contacts were made by the researcher during visits to the neighbourhood. Selection for the qualitative study was based on providing theoretical insights rather than broader generalisations, as noted by Gardner and Chapple[27]. 'Participant' is here defined as residents who both received the *Well London* activities, and volunteered in their delivery.

---

[1] Pseudonyms are used throughout for places and people

For peer review only - http://bmjopen.bmj.com/site/about/guidelines.xhtml

For the post-intervention interview, a total of 45 agreed to a second interview. Reasons given by the 16 who did not attend this second interview were as follows: moved out of the neighbourhood (2), refusal of a follow-up interview (3), no response elicited (9), and illness (2). New recruits were not sought as changes over the intervening period would not have been captured.

Ethnicity, age and length of time in the neighbourhood among the study population were mixed across all three neighbourhoods; each neighbourhood showed variation according to all these categories, most noticeably, ethnicity. It is beyond the scope of this paper to examine the effects of this in detail, other than to recognize this as a difference requiring further investigation.

Table 1. Participant and non-participant profiles

1st Round interviews	Age range	Ethnicity	Gender	*Well London* participation	2nd Round interviews
Hartfield	16-25: 3<br>26-35: 8<br>36-45: 3<br>46-55: 5<br>56-65: 2<br>66-89: 1	African 13, Indian 3, Bangladeshi 1, White British 3, European (Lithuania) 1	Female: 16<br>Male: 5	Participants: 13<br>Non-participant: 8<br><br>Total: 21	Stage 2.<br>P=11<br>NP=8<br><br>Total: 20
Eastford	16-25: 4<br>26-35: 4<br>36-45: 2<br>46-55: 4<br>56-65: 3<br>66-75: 3	Bangladeshi 5, Indian 1, Pakistani 1, Caribbean 2, Black British 1, African 3, Chinese 2, White British 3, Irish 2	Female: 18<br>Male: 2	Participants: 11<br>Non-participants: 9<br><br>Total: 20	Stage 2.<br>P=7<br>NP=8<br><br>Total: 15
Mountside	16-25: 5<br>26-35: 3<br>36-45: 4<br>46-55: 3<br>56-65: 3<br>66-75: 2	White British 5, British Asian 5, Caribbean 4, European (Turkish) 3, African 2, Chinese 1	Female: 11<br>Male: 9	Participants: 10<br>Non-participant: 10<br><br>Total: 20	Stage 2.<br>P=6<br>NP=4<br><br>Total: 10

Data collection and analysis

Pre- and post intervention interviews used the same topic guide and focused on participants experiences of the *Well London* interventions and any reported changes to eating, exercise and mental health practices (see supplementary file). Also both participants and non-participants were asked for their views about the neighbourhood environment. Interviews were recorded and transcribed before being entered into Nvivo. Each transcript was checked for quality, coded and analysed using a framework based on Spencer, Ritchie and O'Conner's 'analytic hierarchy'[28]). This allowed systematic analysis of the large dataset but was flexible enough to allow refinements to the coding. Codes from interviews were identified and grouped under categories generated from the interview topics. Data were analysed not only deductively from the primary outcome measures (changes to healthy eating, physical activity and mental well-being), but also inductively from emerging themes identified from the interviews.

Observational data of the neighbourhoods was recorded both in photographs and written notes and included the local geography, amenities and range of community facilities and activities that were available. A separate researcher was employed at each stage of the study;

For peer review only - http://bmjopen.bmj.com/site/about/guidelines.xhtml

one researcher for stage 1 interviews, and the second researcher for stage 2. They each also conducted the observations simultaneous to the interviews.

Interview quality assurance and ethics

Quality assurance procedures were undertaken to minimise researcher bias when coding the interviews by randomly selecting three interviews, which were then recoded by two independent researchers blind to the initial coding. The three interviews were compared to identify new codes and establish a degree of consensus in applying a particular code to similar text. The University of Westminster Research Ethics Committee approved the study. Following initial telephone/email contact, written consent was obtained from every participant/non-participant. Each individual also received a short pamphlet describing the *Well London* project and an explanation of the qualitative component. Verbal explanation was also provided at the start of the interview process.

The results of the qualitative study are presented here by area. The reason for this is that the context and environment in which participants and non-participants were living and into which the *Well London* interventions and activities were introduced, has been shown to be a key factor in showing why individuals participated. Subsequently, data are presented by area, not theme.

RESULTS

Findings from the qualitative data show participants describing positive changes, both to their individual health and experiences of their neighbourhood as a result of participation in the *Well London* activities. However, equally significant was the degree of variation in how these changes were perceived between each neighbourhood, which was modulated by: mode of delivery, characteristics of individuals, neighbourhood history and attitudes to social interaction. As a consequence, each area is described separately in the results and the basis for using quotes is to represent what was said in relation to the themes.

Overall, participants identified the importance of social interaction as a crucial component of participation in the *Well London* activities. For example: a social gathering that included eating healthy food; gardening and opportunities to chat over a cup of tea; women feeling safer when sharing an evening walk together. Participation in practical health-related activities were only beneficial within a social context. By comparison, non-participants, despite their individual attempts at improving their own health, experienced no benefits either from efforts to change eating or physical activity levels, or from being around others in the community who were participating in *Well London* activities:

> *'So I was left a very lonely bunny for quite a while. I don't like going out walking all the time on my own, I don't like going swimming on my own. I love to do it, but I don't like doing it on my own. If there was a group going, I would go'* Mary, age 48, Irish, non-participant

Furthermore, a small number of non-participants felt excluded from the *Well London* interventions, suggesting there may be some non-beneficial effects. For example, in response to a question about positive changes on the estate as a result of *Well London*, one resident commented:

> *'Well, that "getting better" is a matter of opinion, because I look on it now as a ghetto. It was an unruly estate before. It has quietened down, but now it's a ghetto. I don't go out anymore, I don't do anything anymore. No. No. And I hardly even*

For peer review only - http://bmjopen.bmj.com/site/about/guidelines.xhtml

talk to people now. I mean, I'll sit out at my doorstep and, you know, a lot of people'll stop and chat to me, but I don't really like it'. Karen, age 54, White British, non-participant

Despite scoring high on the 'Indices of Deprivation'[29], the three *Well London* intervention neighbourhoods will be described separately in acknowledgement of their diversity and to bring out the nuances of how place impacts participation and any consequent outcomes.

HARTFIELD is a large housing estate built in the 1950s, comprising low-rise blocks constructed around a series of rectangular grassed areas. Although the most homogenous of the three neighbourhoods in terms of population and environment, prior to *Well London* it was socially fragmented with a dearth of community activities.
Pre-intervention descriptions of Hartfield included:

'And the word 'Hartfield' put horror – it was notorious. Everybody who was difficult was dumped here'. Margaret, 59, White British

It's a dumping ground. It always has been a dumping ground. You know? I begged not to be put on there. I've been there 21 years. Liz, 48, Irish

I will tell you straightaway there was no life in the community before the arrival of Well London. *No, that is the summary of the whole thing; where you are living in an area where there was no life.* Clifford, 46, African (Uganda)

Post-intervention, Hartfield respondents reported the most substantive change in experienced health benefits of all 3 neighbourhoods. Factors that facilitated this included: a) a pro-active, charismatic Well-London coordinator; b) increased safety following changes in policing methods on the estate, instigated by the coordinator; c) a high number of proactive volunteers; and d) residents as stakeholders through the estate's Residents Committee, set up by *Well London*. Benefits described included: enhanced feeling of social cohesion, new knowledge about health, involvement in estate-wide activities, improved relations with neighbours, less complaints about the neighbourhood's lack of cleanliness, safety and violence (See box 1; social interaction).

Box 1 Hartfield	Reported benefits in HE, PA, MHWB, and Social Interaction
**Healthy Eating**	➢ *So you know, after walk we have this exercise to stretch ourselves, and then after that we used to have fruit. Yeah, so we used to sit in the park and we used to eat fruit and that's how I learn to eat fruit basically.* Priya, 34, Indian<br>➢ *Oh my God, people are healthier now. It's changed, it's completely changed. I say that it's changed because I am involved - I know how much to my own particular health* (and*) the health of my family and how much has changed. I'm able to know more now, I know what to eat, what not to eat.* Thomas, 45, African (Ghana)

For peer review only - http://bmjopen.bmj.com/site/about/guidelines.xhtml

**Physical Activity**	➢ *Like before, you know, I used to find walking was kind of one of the painful things, yeah, I wouldn't bother to walk, I would rather take bus rather than walking, but now I feel like, now - rather than taking bus or anything, let's just walk, it's not going to take me that long.* Sandra, 43, African (Uganda)<br>➢ *And we have some people who want to go night walk - like the Somalians. If the place is dark, they would like to walk. Because of, you know, night-time you can also wear your trousers - so that they can walk faster.* Joyce, 38, African (Nigeria)
**Mental Health, Wellbeing**	➢ *The fact that it's made me proud of myself and the whole project and the whole community, because it's made people come in to do the activities.* Bernard 42, African (Ivory Coast)<br>➢ *It has made the community come together, that's what I've seen anyway, people have come together, which is very good.* Claudette, 37, African (Sierra Leone)
**Social interaction**	➢ *Yes we used to be on our own, nobody say hello to each other, but because of Cheryl* (*Well London* co-ordinator), Well London *came to this place* (and) *it start connecting us.* Lorraine, 39, African (Uganda)<br>➢ *Yeah the police, which are responsible to this area, yes it's changed a lot because now they can say 'hello' to you. Sometimes even the kids, if they see them playing outside they will stand and speak with them, and ask them 'are you with elderly adults or are you alone?' And so forth and we are happy for that.* Margaret, 41, African (Ghana)<br>➢ *I think that it does a good thing -* Well London *came to help out. We did a food basket with five foods, I did that as well so. I know the women come to do, they have sewing classes, and it's just - but it's for the coming together, the community together, that's what I think.* Clara, 42, Bangladeshi<br>➢ *I've been proud to say that this is one of my proudest periods in regards to this community. Yeah, this is because our efforts that has been put in place by* Well London *and followed by* Well London *volunteers. Last year I was just a volunteer to* Well London *but this year I am the chairman of* Hartfield Estate *- the estate now compared to what it was in the past, it's a name at least to be proud of.* Frank, 39, African (Nigerian)<br>➢ *Before we started living here I heard (that) the estate wasn't really nice, it wasn't really good. Yeah, in terms of gangs and all those things. But I was, well initially I was a bit scared…no this place has changed now. It's not like the way it used to be before* (Well London)*, there's a lot of cameras around, and then there's this local police office behind, just around there, which is really good, so.*

EASTFORD has undergone extensive regeneration over the last decade, including funding to develop community projects that promote health. This had generated an ethos of community participation and differentiating the *Well London* interventions from these other activities was subsequently more difficult, especially its effects on Mental Health and Well-being. Despite this, the positive changes experienced here by participants refer specifically to the *Well London* activities.

Pre-intervention, Eastford was already defined as a place where things happened: 'Eastford *is great – there's so much to do here!'* Jermina, 26, Bangladeshi.
Post-intervention, respondents experienced some change. Benefits included a) a sense of autonomy from volunteering and involvement in managing and running activities; b) feeling productive and useful; c) increased knowledge of food/cooking & improved health; and c) enhanced feeling of social cohesion.

Box 2 Eastford	Reported benefits in HE, PA, and MHWB and Social Interaction
**Healthy Eating**	➢ *Yeah, whereas say somebody comes in on Tuesday and does a little bit of cooking - it's quite quick, but with the 'Cook & Eat' it was more in depth and they explained things better and you could ask questions and all things like that, yeah, it was much better.* Clare, 38, White British<br>➢ *Earlier I used to be like, junkie foods eating; crisps and all those things. Now it's like more fruit and vegetables and salad in my diet.* Shubha, 28, Indian
**Physical Activity**	➢ *It was great, it's fantastic - I cannot express how good it is to get in there and get your hands dirty, and to see everybody else doing the same thing.* Sarah, 34, White British<br>➢ *Yes, I do a lot, because I'm doing them exercises it's helped me, it's good for my health, I feel much better, I can breathe properly. And you make friend. Yeah, it's good for me - I go out, and you meet friends.* Tricia, 72, Caribbean
**Mental Health and Well-being**	➢ *I feel I can keep my mind going and I feel like my mind has to be active because I don't want to sit down and get depressed or something. If I think bad things then I won't be doing nothing and I don't want to go like that yeah.* Maureen, 48, Irish
**Social interaction**	➢ *I feel so much more confident that we can make this move on; the thing we were given was confidence building. I think that sort of confidence building was something I didn't see – yeah, running an organisation, running that level of budgeting and planning.* Michael, 50, White British<br>➢ *You can see it, just a healthier lifestyle: people busy all the time, people - not so much arguments and you see that less and people are a lot more sociable as well.* Pat, 36, Black British

MOUNTSIDE

For peer review only - http://bmjopen.bmj.com/site/about/guidelines.xhtml

Mountside is a neighbourhood of contradictions, characterized by a geographically dispersed, ethnically and socio-economically diverse but transitory population and a reverse trend in terms of regeneration:

> *'It was a transient population so you'd get people move in for three months, as I say, trash the place or do whatever'*. Paula, 45, White British

> *'It became what I can only describe as a dumping ground for literally anybody. There was no perspective on who was living where and next to whom; people were just thrown into the flats regardless of background, criminal intention or anything'.* Mohan, 52, Indian

> *'When we first moved here it was gorgeous. Oh, you couldn't have wished for a more idyllic place to live. It was quiet, it was flowers, it was lovely neighbours. But of course, a lot of our neighbours then had been here since the block went first up in the '60s, so they were all getting old and consequently all started to die and then their families sold the flats to housing associations. And you go from there'.* Monica, 62, White British

The loss of facilities such as a local cinema, shops and other community activities resulted in the main street consisting of fast food outlets and budget shops – high street brands that used to exist had moved away, apart from a large supermarket. There were pockets of privately owned terraced housing divided from local authority tower blocks, marking a clear socio-economic boundary. Attitudes to *Well London* were similarly divided; some viewed the interventions positively, and some not.

Post-intervention, Mountside respondents recounted little change; positive change was commented on only in relation to the Mental Health and Well-being activities. Factors that prevented change were: a) lack of effective, coordinated local leadership; b) dispersal and transience of the local population; c) lack of cohesive environmental planning; and d) strong sense of neglect and 'being forgotten' by residents.

Box 3 Mountside	Reported benefits in HE, PA, and MHWB
**Healthy Eating**	➢ *Yeah, you know children like chips, sausages, yeah. Just sometimes I'm cooking chips - every time Turkish foods; rice yeah. You know, my older one all the time she wants outside, McDonalds, chicken, chips, she's eating too much. And everywhere this food. I'm telling her 'you know, too much oily inside, you no eat' and she's not listening to me.* Hanife, 36, Turkish<br>➢ *You can see the higher fast food intake, zero exercise, high alcohol and stressful kind of lifestyles that people lead. And this is also supported by the number of fast food outlets that thrive in these areas.* Mohan, 31, Asian British
**Physical Activity**	➢ *We've got one park over the road but, again that's a dangerous place. We've had murders over there, we've had people killing the swans to eat and people sleeping rough over there. So of course, parents weren't taking their kids over to the park, and you can't blame them, I wouldn't go over there.* Marie, 46, White British

For peer review only - http://bmjopen.bmj.com/site/about/guidelines.xhtml

**Mental Health and Well-being**	➢ *It was fantastic, it brought up a lot of issues and a lot of practices and things that I'm already aware of, and I really, really enjoyed it'.* Saroja, 27, Asian British<br>➢ *Oh yeah, and I wish it could continue, I really, really do, because I think it's started to actually break down a few barriers. We were all really sad when it ended and I thought; this is something that could really build up. And I just wish we could have* Well London *permanently. It was a really nice thing, and because it came to this area it made us think, well we are important, it's come here. I know it came here because we were a deprived area but people are listening to us. They're trying to do something to help us. And like I say, the worst thing is that we haven't got it* (now)*. If you can bring it back I'd be ever so grateful and so would a lot of other people.* Karen, 41, White British<br>➢ *I came away having learnt a lot more about the other women – appreciating them more, yeah, I think that's word should be put in there; appreciating other people, not just cultures but people themselves*. Molly, 45, Caribbean
**Social interaction**	➢ *It takes the form of exercise when I can be bothered. I will say I'm a bit lazy sometimes, so you'll do it and then it's like you don't want to take it on, on your own, so you do need motivation* Jan, age 36, White British

CONCLUSION

Participants described an overall positive impact from the *Well London* project activities, but the data also reveal a complex and nuanced picture of if and how outcomes were achieved with two key findings. Firstly, it shows how neighbourhood-level changes did not lead to benefits amongst those who did not participate in project activities. Secondly, the characteristics of neighbourhoods, both social and physical, were fundamental in moderating whether people participated, the nature and extent of the consequent benefits, and any reported changes in health practices. Therefore, participation is dependent on the provision of particular elements that support it; namely a socially cohesive environment in which to get to know neighbours; a safe environment that is well-maintained; access to affordable, nutritious food; a degree of autonomy that allows residents to be involved in decision-making and thereby improve confidence and self-esteem. These findings are substantiated through the statements of participants and shown throughout, in comments concerning the importance of friendships made, improvements to eating habits, and increased feelings of safety, post *Well London*.

The role of the *Well London* co-ordinators also emerged as an important theme across the three areas. With their active involvement through co-ordinated organisation of volunteers, a commitment to the area shown by their understanding of local issues, participation was more successfully implemented. In Hartfield for example, residents frequently cited their co-ordinator; as a 'boundary crosser'[30], she was pivotal in encouraging and facilitating their involvement in activities. As a she By contrast, co-ordination of the activities in Mountside were deemed problematic by many - apart from those attending DIY Happiness groups, which were identified as positive because they acknowledged residents' sense of deprivation. Activities that did less well were those deemed to be out of touch with local needs i.e 'fun'

For peer review only - http://bmjopen.bmj.com/site/about/guidelines.xhtml

activities were less successful than those seen to have direct relevance and benefit, such as stress management. In Eastford, residents were encouraged to lead projects and be involved in decision-making and subsequently, external *Well London* leadership and co-ordination was mentioned less, whereas the benefits of taking a leadership role were spoken of frequently.

Well-being was a central requirement for the exercise of personal agency, which in turn enabled participation in the *Well London* activities. Well-being was tied to factors such as being able to live in a socially cohesive and safe neighbourhood where neighbours respected one another and where problems were recognised and acted upon by local authorities. Once engaged there was an apparent feedback loop whereby further enhancement of well-being increased personal agency and lead to increased involvement in the activities which then lead to changes in attitudes and practices in eating, exercise and mental health. Participants' well-being, agency and participation also interacted with their sense of place, again in an iterative fashion. Following improvements to the physical environment, such as direct involvement of local police in providing safer spaces, further enhancement of well-being and agency were described. Well-being in this instance, appears to be a crucial mediator between agency, participation and improved health practices. A recent review of individual experiences of community engagement also found that active participation in community initiatives has important psychosocial benefits for participants that include enhanced feelings of personal confidence and self-esteem, as well as enhanced social relationships and social cohesion within a community[31, 32].

Comment [JD1]: Sentence removed here

In this study, participation was not a simple binary variable and quantitative measures alone did not pick up the subtleties and complex variations. Our findings show that participation is a complex and dynamic process with well-being at its core, acting as a catalyst that enables participation through a related sense of personal agency. Through further enhancement of well-being and associated social cohesion, improvements in health practices were experienced, just as in its absence no benefits were recounted. However, participation was not universally desired; some reported feeling excluded from the *Well London* interventions and the subsequent changes taking place in the neighbourhood (see participant comment, pg 6), whilst others reported little interest in taking part because improving health was neither a priority nor an personal goal.

These findings also confirm that health practices are not a separate 'capsule' of behaviour[33, 34], but are embedded within particular social, cultural and physical milieus. Across the three neighbourhoods however, there was a clear gradient of change with the greatest change seen in the presence of; a) involvement and support of external organisations; b) personal and collective agency enhanced by effective leadership in project activities; c) social cohesion fostered by *Well London* activities. Where the physical and social environment remained unchanged, there was less participation and therefore fewer benefits. Also, each area reflected considerable variation in levels of maturity and self-management: each was graded in terms of what progress was possible, Mountside being at the beginning of area level change with safety and environmental pollution still an issue in contrast to Eastford, which featured a more developed and progressive attitude due to investment both financial and from the local authority. Hartfield was in the midst of significant degrees of change through both necessity and a relatively recent influx of enthusiastic residents supported by an equally enthusiastic co-ordinator.

Comment [JD2]: Sentence removed here

As others have identified, the dynamics of participation from the perspective of individual agency have been neglected[17, 18, 32]. In addressing this, our findings show participation as a dynamic and flexible process with agency at its core. Also, the community engagement approach fed back into and reinforced feelings of well-being and agency and thus encouraged

For peer review only - http://bmjopen.bmj.com/site/about/guidelines.xhtml

and supported changes in health practices. The findings are consistent with elements of Popay's[17] proposed pathways by which community engagement leads to health outcomes, and specifically that social capital/cohesion and community empowerment are important intermediaries[17]. Additionally they point to the need for further understanding of how these interact with agency, well-being and empowerment at the individual level and in different social contexts, in order to achieve inclusive engagement with such programmes. As Popay argues[25], people's own ideas need to be incorporated fully in the design and delivery of proposed health interventions.

Acknowledgements We thank all of the study participants for giving generously of their time; the project delivery partners for their assistance; our colleagues at the University of East London, University of Westminster and the London School of Hygiene and Tropical Medicine for their support and cooperation.
Author contributions: J Derges data collection, interpretation, analysis and writing; S Jain, data interpretation and analysis; A Clow, data analysis and interpretation; R Lynch, data collection, data analysis; A Renton, project lead; A Draper, data interpretation, analysis and writing.
Funding: This work was supported by the Wellcome Trust, Grant code: 083679/Z/07/Z
Competing interests: None
Ethics approval: granted by University of Westminster Ethics Committee
Data sharing statement: Anonymised interview transcripts may be obtained on request from Dr A. Draper, University of Westminster

REFERENCES

1. WHO: **The Jakarta Declaration on Leading Health Promotion into the 21st Century**. Fourth International Conference on Health Promotion, Jakarta, 1997.
2. Marmot M: **The Marmot Review: Fair Society, Healthy lives – Strategic Review of Health Inequalities in England post-2010**. London, 2010.
3. Whitehead M: **A typology of actions to tackle social inequalities in health**. Journal of Epidemiology and Community Health 2007;**61:**473-478.
4. Anderson A: **The Community Builder's Approach to Theory of Change: A Practical Guide to Theory Development**. New York: The Aspen Institute Roundtable on Community Change 2005.
5. Bryce J, Victora CG, Habicht JP, Black RE, et al. (MCE-IMCI Technical Advisors): **Programmatic pathways to child survival: results of a multi-country evaluation of**

For peer review only

Integrated Management of Childhood Illness. Health Policy and Planning Suppl 2005;**1:**15-117.

6. O'Dwyer LA, Baum F, Kavanagh A, MacDougall C: **Do area-based interventions to reduce health inequalities work? A systematic review of evidence**. Critical Public Health, 2007;**17**:317-335.
7. Victora CG, Habicht J-P, Bryce J: **Evidence-based public health: moving beyond randomized trials.** American Journal of Public Health 2004;**94**:400-405.
8. Medical Research Council: **A framework for development and evaluation of RCTs for complex interventions to improve health.** London: MRC; 2000.
9. Medical Research Council: **Developing and evaluating complex interventions: new guidance.** London: MRC; 2008.
10. Hawe P , Sheill A, Riley T, Gold, L: **Methods for exploring implementation variation and local context within a cluster randomized community intervention trial**. Journal of Epidemiology and Community Health 2004;**58**:788-793.
11. Ogilvie D, Cummins S Petticrew M, White M, Jones A, Wheeler K: **Assessing the evaluability of complex public health interventions: five questions for researchers, funders, and policymakers.** Milbank Q 2011,**89**:206-225.
12. Hawe P, Shiell A, Riley T: **Complex interventions: how "out of control" can a randomized controlled trial be?** BMJ 2004, **328**: 1561-1563
13. **Well London: communities working together for a healthier city**. [http://www.welllondon.org.uk]
14. Draper AK, Hewitt G, & Rifkin SB: **Chasing the Dragon: developing indicators for the assessment of community participation in health programmes**. Soc Sci Med 2010,71:1102-1109. DOI 10.1016/j.socscimed.2010.05.016

For peer review only

15. Milton B, Attree P, French B, Povall S, Whitehead M, Popay J: **The impact of community engagement on health and social outcomes: a systematic review**. Community Development Journal 2012;**47**(3): 316-334.

16. Preston R, Waugh H, Larkins S, Taylor J: **Community participation in rural primary health care: intervention or approach?** Australian Journal of Primary HealthCare 2010;**16**:4-16.

17. Popay J: **Community engagement and community development and health improvement: a background paper for NICE**. London: NICE 2006.

18. Wallerstein N: **What is the evidence on effectiveness of empowerment to improve health?** Copenhagen, Denmark, WHO Regional Office for Europe, 2006. Health Evidence Network report; http://www.euro.who.int/Document/E88086.pdf. (accessed January 2013)

19. Cleaver F: **Institutions, agency and the limitations of participatory approaches to development**. In: Hickey S, Mohan G, eds. Participation: From Tyranny to Transformation? London Zed Books, 2004:271-283.

20. Egan M, Tannahill C, Petticrew M, Thomas, S: **Psychosocial risk factors in home and community settings and their associations with population health and health inequalities: a systematic meta-review.** BMC Public Health 2008;**8**:239

21. Forde I, Raine R: **Placing the individual within a social determinants approach to health inequity**. The Lancet 2008;**122**(11):1177-1187.

22. Wall M, Hayes R, Moore D, Petticrew M, Clow A, Schmidt E, Draper A, Lock K, Lynch R, Renton A: **Evaluation of community level interventions to address social and structural determinants of health: a cluster randomised controlled trial**. BMC Public Health 2009;9:207 DOI:10.1186/1471-2458-9-207.

23. Petticrew M: **When are complex interventions 'complex'? When are simple interventions 'simple'?** Euro J Public health 2011;**21**:397-8.

For peer review only - http://bmjopen.bmj.com/site/about/guidelines.xhtml

24. Phillips G, Renton A, Moore DG, Bottomley C, Schmidt E, Lais S, Yu G, Wall M, Tobi P, Frostick C, Clow A, Lock K, Petticrew, Hayes R: **The Well London program – a cluster randomized trial of community engagement for improving health behaviors and mental well-being: baseline survey results.** Trials 2012 Jul 6;13:105.doi: 10.1186/1745-6215-13-195.
25. Popay J: **Whose theory is it anyway?** Journal of Epidemiology and Community Medicine 2006;**60**:571-572.
26. Patton MQ: **Qualitative Research and Evaluation Methods**. 3rd Edition. Sage Publications, Thousand Oaks, California 2002:236.
27. Gardner K, Chapple A: **Barriers to referral in patients with angina: a qualitative study**. BMJ 1999;319-321.
28. Spencer L, Ritchie J, O'Connor W: **Analysis: Practices, principles and processes**. In *Qualitative research practice: a guide for social science students and researchers.* Edited by J. Ritchie & J Lewis. Thousand Oaks, CA: Sage 2003:200-218.
29. **English indices of deprivation 2010 Government Report** http://www.communities.gov.uk/publications/corporate/statistics/indices2010
30. Clow, A Hamer, M: **The iceberg of social disadvantage and chronic stress: implications for public health.** Neurosci Biobehav Rev 2010;**35**:1.
31. Attree P, French B, Milton B, Povall S, Whitehead M, Popay J: **The experience of community engagement for individuals: a rapid review of the evidence**. Health and Social Care in the Community 2011;**19**:250-60.
32. Polgar S. **Health and human behaviour: areas of interest common to the social and medical social sciences**. Current Anthropology 1962;**3**:159-205
33. Fienieg B, Nierkins V, Tonkens E, Plochg T, Stronks K. **Why play an active role? A qualitative examination of lay citizens' main motives for participation in health promotion**. Health Promotion International 2012;**27**:416-26.

For peer review only

For peer review only - http://bmjopen.bmj.com/site/about/guidelines.xhtml

34. Kilpatrick S, Cheers B, Gilles M, Taylor J: **Boundary crossers, communities, and health: exploring the role of rural health professionals**. Health Place 2009;**15**:284-290

For peer review only

For peer review only - http://bmjopen.bmj.com/site/about/guidelines.xhtml

UoW Well London Evaluation
Topic Guide for Phase 2 Interviews

Neighbourhood

- **Can you tell me about this area?**
  - What is it like living here?
  - Do you like living here?
  - Who lives here?
  - What are your neighbours like?
  - How did you come to be living in this area?

- **How long have you been here?**
  - Where were you before?
  - How has this area changed since you've lived here?
  - What sense of belonging or attachment, if any, do you feel to the area?
  - Do you see this area as "home"?

- **Is there a lot going on in this area?**
  - What goes on?
  - Who goes?
  - Why do people go/not go?
  - What, if anything, do you participate in in the local area or community?
  - Why do, or why don't you participate in activities in the local area?
  - Do you socialise/have friends locally?

- **How safe and comfortable do you feel in this area?**
  - What are the good things about living here?
  - Do you worry about the area?
  - Have you experienced any problems with the area or the local community?

- **How healthy are people around here?**
  - Are people locally concerned about their health?
  - Is it easy to be healthy here? Do you have access to health activities?
  - Are there things about living in this area that you think are unhealthy?
  - How do you think living in this area affects your health and well being?

Health and Wellbeing

- **What are the features to being healthy?**
  - What is a healthy lifestyle?
  - Would you say you are in good health?
  - Would you say you have a healthy lifestyle?

For peer review only

UoW WL Evaluation
Revised Interview Topic Guide
18/02/10

- **Do you think about your health very much?**
    - What, if anything do you do to keep yourself healthy or improve your health?
    - Do you worry about your health?
    - What do you do about this worry about your health?

- **How much do you worry about things generally?**
    - Do you have a lot to worry about?
    - What do you do when you worry?
    - How optimistic or negative do you think you are as a person?
    - How does this outlook affect the way you live your life do you think?
    - What are the positive and negative things in your life?

- **Are there people you can trust and talk to if you have problems or worries?**
    - Do you have much contact with these people?
    - Do they live nearby?

<u>Well London</u>

- **Have you heard of the Well London Project or know of any Well London activities in the area?**

- **Have you participated in any Well London activities (including the community cafes)?**
    - If so, what was your experience of these?

- **Have you felt there has been any benefit to yourself or your area from the Well London Project?**

For peer review only
